# Interaction of TFAP2A with the Ku70/80 complex is crucial for HIF‐dependent activation of hypoxia‐inducible genes

**DOI:** 10.1111/febs.70025

**Published:** 2025-02-24

**Authors:** Amalia Kanoura, Antonis Giakountis, Chrysa Filippopoulou, Angeliki Karagiota, George Stamatakis, Martina Samiotaki, George Panayotou, George Simos, Georgia Chachami

**Affiliations:** ^1^ Laboratory of Biochemistry, Faculty of Medicine University of Thessaly Larissa Greece; ^2^ Department of Biochemistry and Biotechnology University of Thessaly Larissa Greece; ^3^ Institute for Bioinnovation, Biomedical Sciences Research Center ‘Alexander Fleming’ Attica Greece; ^4^ Gerald Bronfman Department of Oncology, Faculty of Medicine McGill University Montreal Canada

**Keywords:** HIF, HIF‐1α, SUMO, SUMOylation, TFAP2A

## Abstract

Hypoxia can be established under pathological conditions, such as cancer, due to the imbalance between oxygen supply and consumption. Hypoxia‐inducible transcription factor HIF‐1 mediates the physiological response to hypoxia but also regulates multiple steps of carcinogenesis. Despite its well‐defined oxygen‐dependent activation, many aspects of HIF‐1 transcriptional activity as well as interaction with chromatin remain elusive. We have recently shown that hypoxia triggered deSUMOylation of TFAP2A. To study the possible role of TFAP2A in the transcriptional response to hypoxia, we performed ChIP‐seq analysis. Our results have now shown that TFAP2A resides together with HIF‐1α on the promoters of a subset of hypoxia‐regulated genes, the mRNA expression of which is downregulated by silencing of *TFAP2A*. Interestingly, CRISPR‐mediated knockdown of TFAP2A expression under hypoxia decreased the occupancy of HIF‐1α on these promoters and affected chromatin accessibility. Mechanistically, we reveal that the Ku70/Ku80 protein complex interacts with deSUMOylated TFAP2A under hypoxia and participates in HIF‐dependent gene expression. Moreover, using stable expression of TFAP2A forms that either lack or constitutively carry a SUMO modification, we could show that SUMOylation affects binding of TFAP2A to chromatin. Overall, our data suggest that TFAP2A is an important co‐regulator of the HIF‐1‐dependent transcriptional response to hypoxia and SUMOylation fine‐tunes this regulation. As both TFAP2A and HIF‐1 play critical roles in cancer progression, a detailed characterization of their crosstalk could lead to novel therapeutic strategies for targeting and killing cancer cells in hypoxic tumors.

## Graphical abstract

Under normoxic conditions, the SUMOylated fraction of TFAP2A cannot interact with the Ku70/Ku80 complex, nor it can bind to chromatin. The unSUMOylated fraction pre‐occupies the chromatin of a subset of hypoxia‐induced genes together with the Ku70/Ku80 complex for basal euchromatin formation. Under hypoxic conditions, the SUMOylation equilibrium shifts towards the unSUMOylated state of TFAP2A. This enhances its binding to DNA and association with the Ku70/80 complex to ensure proper HIF binding, making chromatin more favorable for transcription.

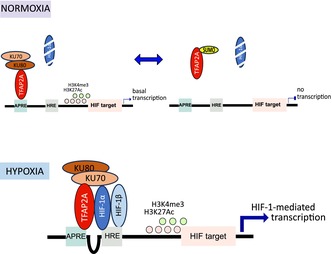

**Abbreviations**
ALDOC: aldolase, fructose‐bisphosphate C
ARNT: aryl hydrocarbon receptor nuclear translocator
ATRX: alpha‐thalassemia/mental retardation X‐linked helicase II or ATP‐dependent helicase ATRX
BIRC3: baculoviral IAP repeat containing 3
CDK8: cyclin‐dependent kinase 8
CDK9: cyclin‐dependent kinase 9
CEBPB: CCAAT enhancer‐binding protein beta
CREB: cAMP Response element‐binding protein
E2F: transcription factor E2F
EMT: epithelial‐to‐mesenchymal transition
ENO1: enolase 1
ERBB2: Erb‐B2 receptor tyrosine kinase 2
FOS: Fos proto‐oncogene AP‐1 Transcription factor subunit
GAPDH: glyceraldehyde‐3‐phosphate dehydrogenase
HIFs: hypoxia‐inducible factors
HREs: hypoxia response elements
ILF2: interleukin enhancer‐binding factor 2
ILF3: interleukin enhancer‐binding factor 3
IRF2BP2: interferon regulatory factor 2 binding protein 2
KCTD15: potassium channel tetramerization domain containing 15
KDMs: lysine demethylases
LDHA: lactate dehydrogenase A
LPIN1: lipin 1
NFRKB: nuclear factor related to KappaB binding protein
NPM1: nucleophosmin
NuRD: nucleosome remodeling and deacetylase
PHDs: prolyl hydroxylases
PHLDB2: Pleckstrin homology like domain family B member 2
PRKDC (or DNA‐PK): protein kinase, DNA‐activated, catalytic subunit (or DNA‐dependent protein kinase)
RPLP1: ribosomal protein lateral stalk subunit P1
SEC: super elongation complex
SERPINE: serpin family E member
SET1B: SET domain containing 1B histone lysine methyltransferase
SUMO: small ubiquitin‐like modifier
TFAP2A: transcription factor activating enhancer‐binding protein 2 alpha
TFAP2D: transcription factor activating enhancer‐binding protein 2 delta
TGFbI: transforming growth factor; beta‐induced
TIP60 (or KAT5): 60 KDa Tat‐Interactive Protein (or Lysine Acetyltransferase 5)
TRIM28: tripartite motif containing 28
VEGF: vascular endothelial growth factor
VHL: von Hippel–Lindau
XRCC5: X‐Ray repair cross complementing 5
XRCC6: X‐Ray repair cross complementing 6
ZBTB38: zinc finger and BTB domain containing 38

## Introduction

Unbalance between oxygen supply and consumption in cells and tissues can result in low oxygen availability, a state also known as hypoxia. Cells initiate a variety of responses to hypoxia, involving changes in gene expression and protein function. The hypoxia‐inducible factors (HIFs) play a central role in the adaptive response to hypoxia as they are the key mediators of the transcriptional response [1, 2]. HIFs are heterodimers consisting of a hypoxia‐inducible HIF‐α subunit (with three known isoforms in human, namely HIF‐1α, HIF‐2α, and HIF‐3α) and a constitutively expressed HIF‐β subunit (HIF‐1β or aryl hydrocarbon receptor nuclear translocator or ARNT) [3]. Under normal oxygen conditions (normoxia), HIF‐α is continuously produced and destroyed, in a process involving oxygen‐dependent modification by prolyl hydroxylases (PHDs), von Hippel–Lindau (VHL)‐mediated polyubiquitination and subsequent proteasomal degradation [4]. Under hypoxia, the PHDs are inactivated due to oxygen unavailability and, subsequently, HIF‐α escapes degradation and translocates into the nucleus, where it forms a heterodimer with HIF‐1β. The HIF heterodimer binds to promoters of target genes, on specific DNA elements called hypoxia response elements (HREs). It has been suggested that HIFs bind at pre‐existing open chromatin loci of target genes, loaded with paused RNA pol II. The recruitment of HIFs is thought to initiate the assembly of high‐order transcription protein complexes, such as the Mediator complex (via associated kinase CDK8) and the Super elongation complex (SEC), in order to phosphorylate and release RNA pol II for subsequent transcription elongation [5, 6]. However, it is not well understood how the open chromatin status of HIF‐target genes is maintained.

Furthermore, critical details of the mechanism of HIF‐dependent transcriptional activation still remain elusive. For example, although HIF‐1 and HIF‐2 can bind to the same HRE sequence, they control unique genes in a cell‐ and tissue‐dependent manner, while little is known on what controls their target specificity [7]. Recent data have also shown that the presence of HREs in hypoxia‐induced promoters is necessary, but not sufficient, to direct gene expression in response to hypoxia [8]. All these suggest that additional transcription factors or co‐regulators are necessary to control HIF specificity and target‐gene selection. Indeed, sequence analysis of regions around the HREs has revealed sequence motifs characteristic of other transcription factors, including FOS, CREB, and CEBPB [9, 10]. Certain of these factors may act as pioneer transcription factors that actively affect transcription by directly opening condensed chromatin or by recruiting other transcription factors and histone modification enzymes. HIF‐mediated transcription at the level of individual targets could be also regulated by post‐translational modifications that locally affect chromatin structure and accessibility. Studies over the last years support that HIF transcriptional activity requires epigenetic factors such as acetyltransferase TIP60, SET Domain Containing 1B Histone Lysine Methyltransferase (SET1B) and lysine demethylases (KDMs). These factors change the epigenetic landscape in favor of transcription suggesting that a functional interplay between chromatin, post‐translational modifications, and DNA binding proteins is needed to support the HIF transcriptional response [11]. As another example, NPM1 (Nucleophosmin) a nucleolar protein, but also a known histone chaperone and chromatin remodeller, has been recently shown to promote the phosphorylation‐dependent recruitment of HIF‐1 to HREs, its interaction with acetylated histones, and high expression of HIF‐1 target genes under hypoxia [12].

We have previously shown that SUMOylation of proteins triggers transcriptional and nontranscriptional responses during adaptation to hypoxia [13, 14]. Specifically, using a SUMO‐IP combined with quantitative proteomics (SILAC), we could demonstrate that hypoxia triggers dynamic changes in SUMOylation of a set of transcription factors or transcriptional co‐regulators such as TFAP2A, ATRX, IRF2BP2, KCTD15, NFRKB, and ZBTB38 without, however, causing concomitant changes in their protein expression levels. We chose to further investigate TFAP2A, the SUMOylation of which was downregulated by hypoxia, because it can physically interact with HIF‐1α and HIF‐2α and stimulated the transcriptional activity of HIF‐1, suggesting a direct involvement in the HIF‐mediated transcriptional cascade [13]. We now provide mechanistic details on the role of TFAP2A in the response to hypoxia, by showing that TFAP2A occupies the promoters of a subset of HIF‐regulated genes and its presence is required for increasing chromatin accessibility and stabilizing the association of HIF‐1 with its target genes leading to their optimal activation. We further provide evidence that TFAP2A SUMOylation regulates both its binding to DNA and its physical interaction with the ATP helicase complex Ku70/Ku80, recruitment of which is important for transcriptional activation.

## Results

### TFAP2A is recruited on promoters of hypoxia‐inducible genes together with HIF‐1

**Fig. 1:**
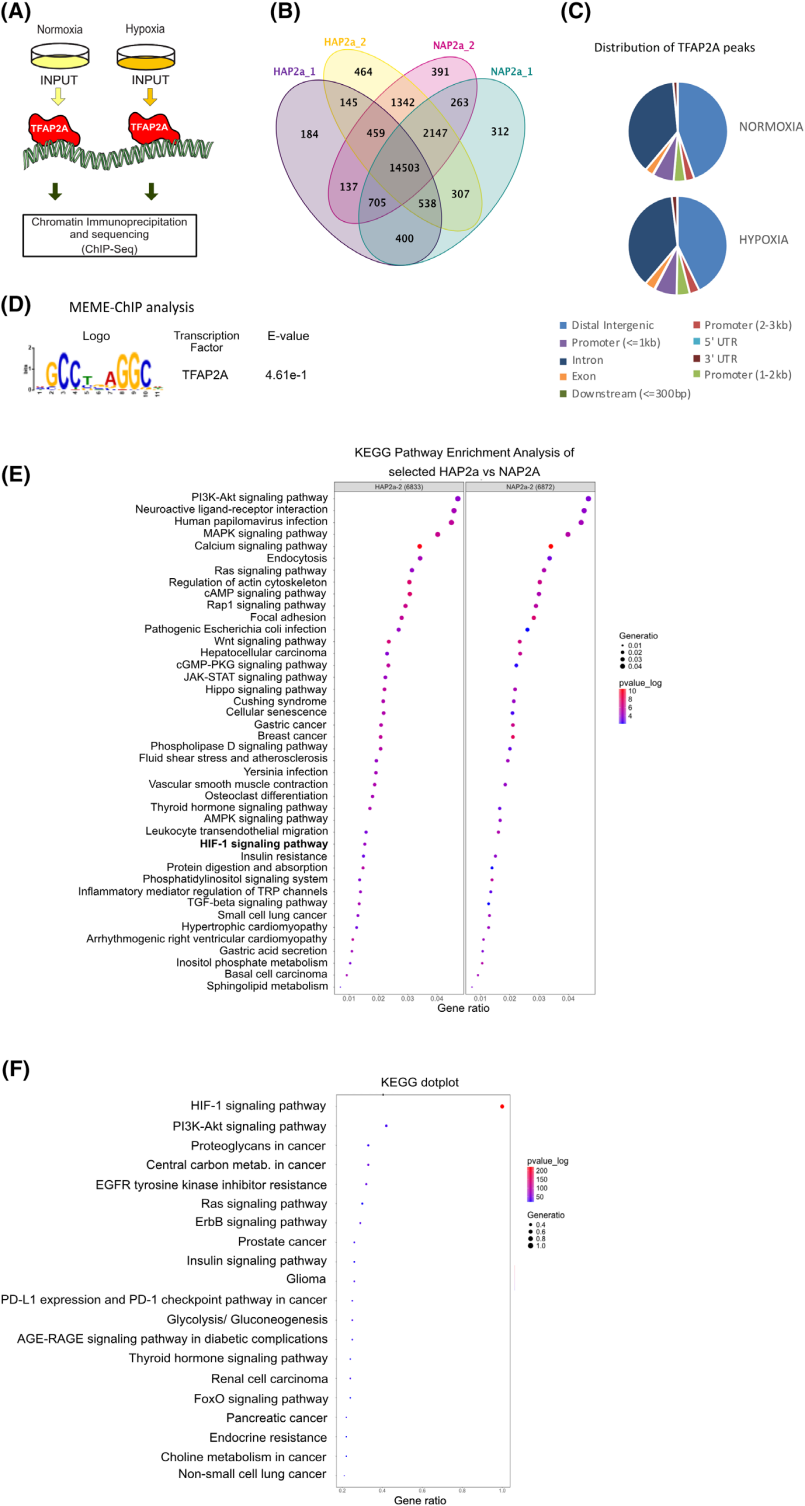
TFAP2A is recruited to promoters of hypoxia‐inducible genes together with HIF‐1. (A) Schematic overview of the experimental procedure (ChIP‐seq analysis). HeLa cells were incubated in normoxia (21% O_2_) or hypoxia (1% of O_2_) for 24 h, and their lysates were subjected to chromatin immunoprecipitation against TFAP2A followed by high‐throughput DNA sequencing (two biological repetitions). (B) Venn diagram representing the number of TFAP2A bound‐loci (peaks) under normoxia (NAP2a) or hypoxia (HAP2a) in two different experiments described in (A). (C) Pie charts representing the distribution of TFAP2A peak annotations on target genes under hypoxia and normoxia. (D) MEME‐ChIP enrichment analysis of unique peaks. Unique peaks are significantly enriched for the TFAP2A motif. (E) Dot plots highlighting the KEGG pathways enriched in TFAP2A peaks in normoxia (NAP2a) or hypoxia (HAP2a). (F) KEGG analysis of TFAP2A peaks found in HIF‐signaling genes.

In order to investigate the mechanism underlying the involvement of TFAP2A in HIF‐1‐mediated transcription, that we previously observed [13], we used chromatin immunoprecipitation followed by DNA sequencing (ChIP‐Seq) as means of identifying direct transcriptional targets of TFAP2A in HeLa cells. HeLa cells grown under normoxia or hypoxia for 24 h were lysed and the lysates were subjected to ChIP using antibodies against TFAP2A followed by high‐throughput sequencing (ChIP‐seq, two biological repetitions) (Fig. 1A). ChIP‐Seq analysis detected 17 618 TFAP2A bound‐loci present in both repetitions under normoxia and 15 645 under hypoxia (File S1). Almost 82% (14 503) of the total peaks identified under normoxia were common between hypoxic and normoxic conditions (Fig. 1B, Venn Diagram, File S1 for details). Under both conditions, the TFAP2A peaks were detected with higher frequency within genes (including introns) than in intergenic regions. Approximately 15% of the TFAP2A peaks were found enriched in the promoter‐TSS regions, around 0–1, and 1–3 kb upstream of the TSS, and this percentage was not significantly altered under hypoxia (Fig. 1C and File S1‐peak annotation distribution). *De novo* motif analysis of the peaks showed that the most significant motif for TFAP2A enrichment is the AP‐RE core sequence, 5′‐*GCCN3GGC*‐3′, (Fig. 1D and File S2), as has been previously described [15, 16]. We used KEGG pathway analysis to link the TFAP2A‐bound genes to cellular processes. As shown in Fig. 1E TFAP2A binding was enriched in genes associated with PI3K‐Akt, Wnt, and TGF‐beta signaling pathway that TFAP2A was reported to be involved [17, 18, 19, 20]. Interestingly, KEGG analysis of our data derived from hypoxic cells showed a significant enrichment of TFAP2A in HIF‐1 signaling pathway genes (Fig. 1E and File S1). These genes are mostly involved in glycolysis, central carbon metabolism, kinase signaling pathways, etc. (Fig. 1F).

To obtain a clear overview of the topological distribution between TFAP2A and HIF‐1 locations within the promoters, we performed a peak relative distance analysis and compared the relative distance (presented on a scale between 0 and 0.5 with values close to 0 indicating spatial correlation of peaks) between the TFAP2A (from our own ChIP‐Seq analysis) and the HIF peaks (from publicly available data [21]) (PRJNA714954) in the HIF‐1 signaling pathway genes identified by our analysis (Fig. 1E) to the pan‐genomic relative distances. We observed that, while there was a rather random distribution between TFAP2A and HIF peaks in all TFAP2A‐bound genes (relative distances scattered between 0.1 and 0.5), TFAP2A peaks were found to be spatially correlated (relative distance < 0.1) to HIF‐1 peaks within the HIF‐dependent genes (File S3 (Fig S1A) and File S1‐relative distance analysis). This observation indicates a probable crosstalk between these two transcription factors for the transcriptional regulation of a common set of hypoxia‐activated promoters.

To better define the genomic overlap of all TFAP2A and HIF‐binding sites, we performed a whole‐genome comparable peak analysis between the TFAP2A binding peaks identified by our experiment and the publicly available ChIP‐Seq datasets of TFAP2A (ENCODE‐PRJNA63447), HIF‐1α, HIF‐1β, and HIF‐2α (PRJNA714954), all derived from HeLa cells [21]. File S3 (Fig S1B) shows an indicative comparative ChIP‐Seq analysis in genome browser (https://genome.ucsc.edu) of two glycolytic genes, namely ENO1 and LDHA that are known HIF‐1 target genes. Interestingly, HIF‐1α/HIF‐2α/ΗIF‐1β and TFAP2A co‐occupy the same region of the ENO1 promoter (overlapping its TSS). In the case of LDHA, a major TFAP2A peak is located approximately 10 178 bp away from the HIF‐binding site and the known HRE showing that TFAP2A could bind to at least a subset of hypoxia‐inducible promoters in close or distal vicinity to HIF‐binding sites.

**Fig. 2:**
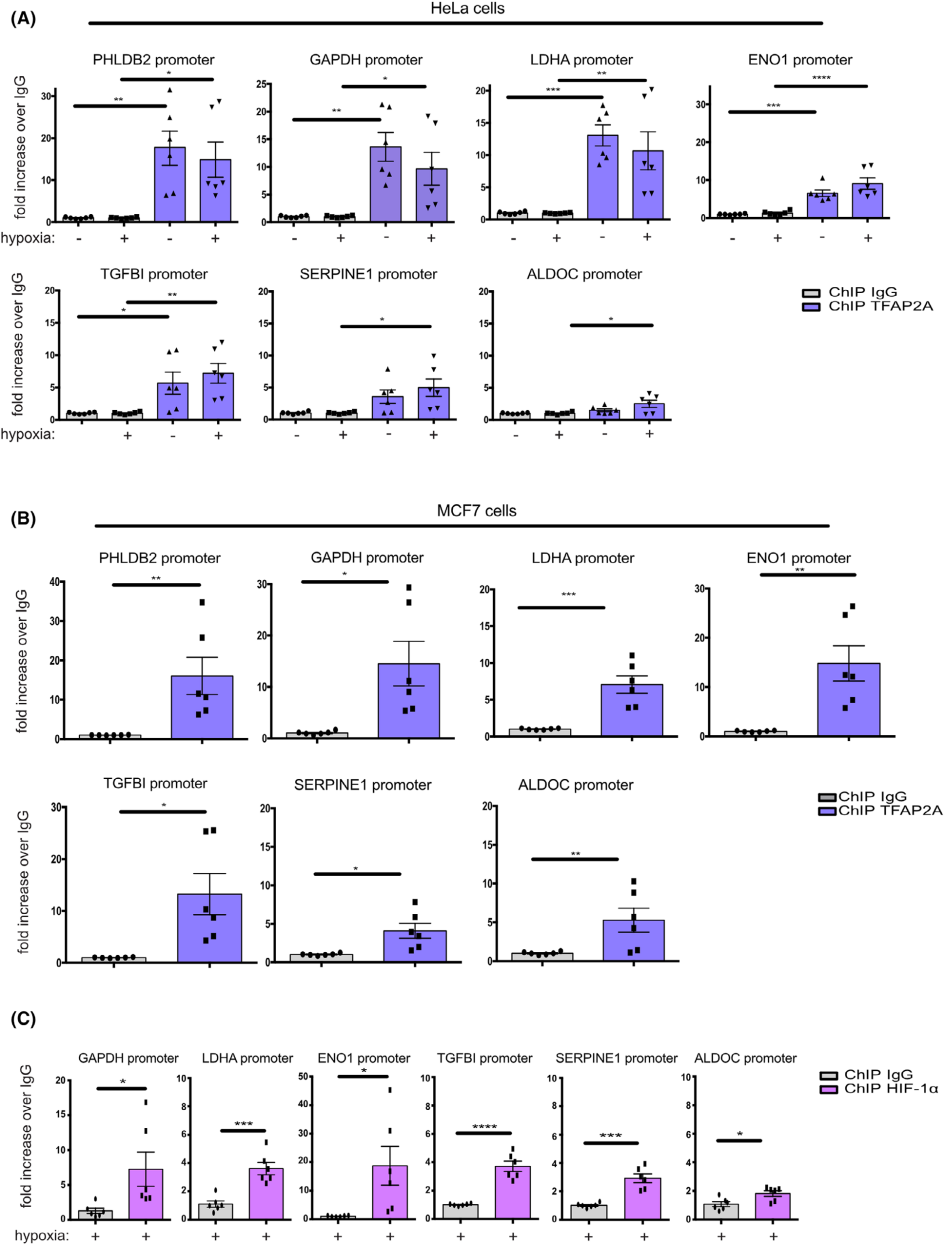
TFAP2A occupies promoters of HIF‐inducible genes both in normoxia and hypoxia. (A) HeLa cells were incubated in normoxia (−, 21% O_2_) or hypoxia (+, 1% of O_2_) for 24 h, and their lysates were subjected to chromatin immunoprecipitation against TFAP2A or rabbit IgG followed by qRT‐PCR analysis, using primers for the indicated promoters. (B) MCF7 cells were incubated under normoxia as in (A), and their lysates were subjected to chromatin immunoprecipitation against TFAP2A or rabbit IgG followed by qRT‐PCR analysis, using primers for the indicated promoters. (C) HeLa cells were incubated as in (A), and their lysates were subjected to chromatin immunoprecipitation against HIF‐1α or rabbit IgG followed by qRT‐PCR analysis, using primers for the indicated promoters. Values in (A–C) are the mean of three independent experiments performed in duplicates −/+ SE and expressed as fold increase compared to each control conditions (ChIP against IgG), normalization was performed against INPUT Ct (*P < 0.05; **P < 0.01; ***P < 0.001, ****P < 0.0001, comparisons were made by one‐way ANOVA Tukey's multiple comparisons).

In order to validate the results of our ChIP‐Seq analysis, we selected six genes (namely GAPDH, LDHA, ENO1, ALDOC, TGFBI, and SERPINE1 that are known HIF/hypoxia targets) [11, 12, 22, 23, 24] that showed strong occupancy by both TFAP2A and HIF according to our ChIP‐Seq data and the comparative ChIP‐Seq analysis, respectively. As a positive control for TFAP2A binding, we used the promoter of PHLDB2 (Pleckstrin Homology Like Domain Family B Member 2) that was shown before to be a direct TFAP2A target gene [15]. ChIP against TFAP2A followed by qPCR analysis revealed a high level of enrichment of TFAP2A in the PHLDB2 promoter but also a significant enrichment in HIF‐targeted promoters, such as the ones from the glycolytic genes GAPDH, LDHA, ENO1, ALDOC as well as TGFBI and SERPINE1, under both normoxia and hypoxia in approximately similar levels (Fig. 2A). Binding of TFAP2A in promoter regions of the same hypoxia‐dependent genes could be also shown in breast carcinoma MCF7 cells (Fig. 2B), suggesting that binding of TFAP2A to HIF‐regulated promoters is not a cell type‐specific event. The co‐occupancy of HIF‐1α on the same promoter loci as TFAP2A under hypoxia was also experimentally confirmed using ChIP against HIF‐1α and qPCR (Fig. 2C). These results strongly suggest that TFAP2A is bound to at least a subset of hypoxia‐inducible promoters, already under normoxia, in close or distal proximity to HIF‐binding sites and could cooperate with HIF‐1 in order to trigger transcriptional activation in response to low oxygen.

### TFAP2A depletion impairs mRNA expression of a subset of HIF‐1‐dependent genes

**Fig. 3:**
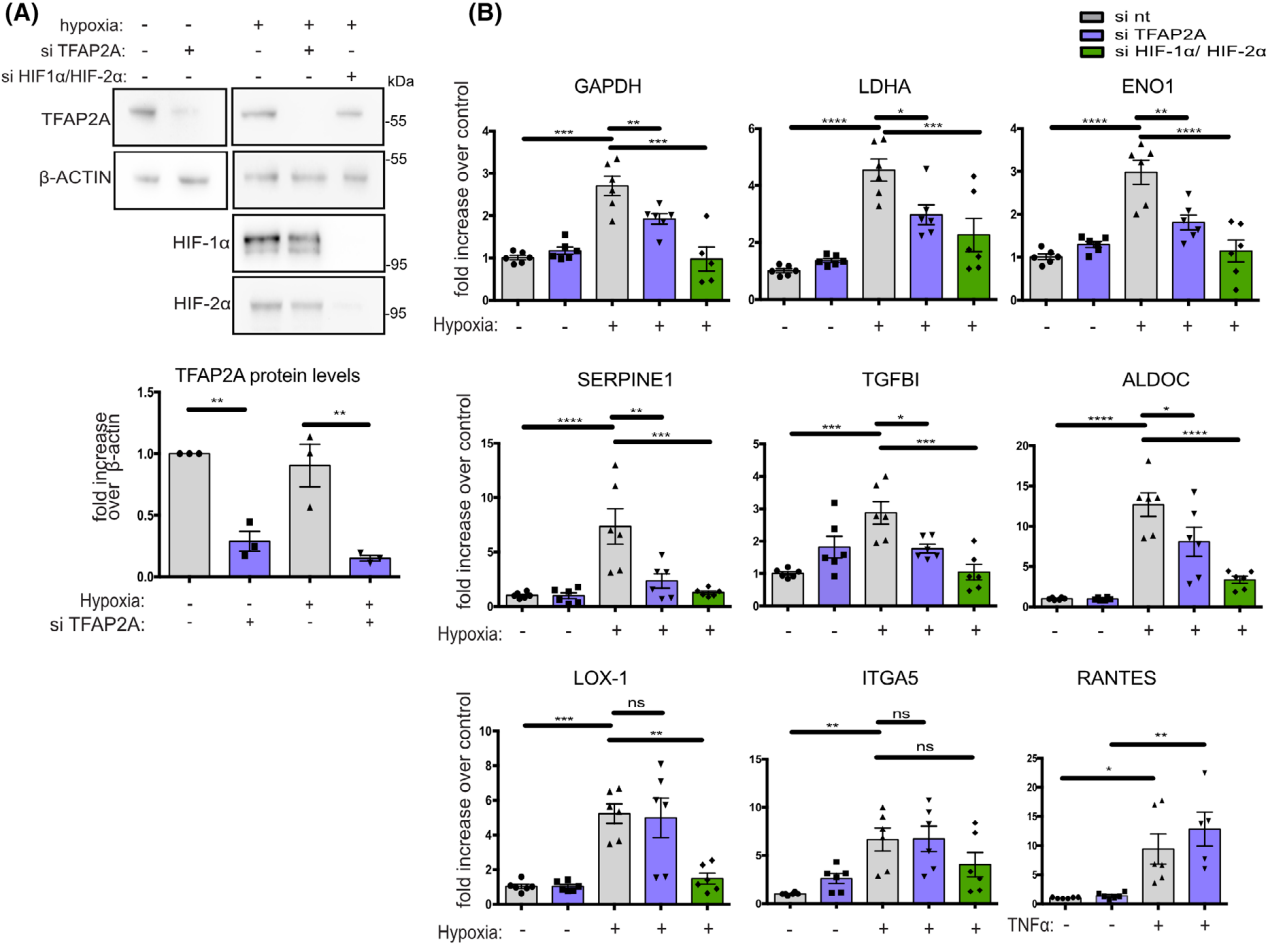
TFAP2A depletion impairs mRNA expression of a subset of HIF‐1‐dependent genes. (A) Immunoblotting analysis of total extracts of HeLa cells transfected with nt siRNA (−), siRNA against TFAP2A or HIF‐1α/HIF‐2α and incubated under normoxia (−, 21% O_2_) or hypoxia (+, 1% O_2_) for 24 h, using the indicated antibodies. β‐actin was used as loading control. The western blot shown is representative of three independent experiments. Position of MW is shown on the right. Quantification of TFAP2A protein levels (from 3 experiments) in the absence or presence of siRNA against TFAP2A is presented in the diagram below. (B) HeLa cells were transfected with nt siRNA, siRNA against TFAP2A or HIF‐1α/HIF‐2α and incubated as in (A) and determination of mRNA levels of the indicated hypoxia and TNFα‐dependent genes, was measured using qRT‐PCR. 18S rRNA was used for normalization. Values are the mean of three independent experiments performed in duplicate −/+ SE and are shown as fold increase compared to normoxic control (−, si nt) (*P < 0.05, **P < 0.01, ***P < 0.001, ****P < 0.0001 comparisons in all diagrams between different groups were made by one‐way ANOVA Tukey's multiple comparisons).

We next examined the role of TFAP2A in the transcriptional activation of the six HIF‐target genes that we previously identified as common TFAP2A and HIF‐1α targets (Figs 1 and 2). TFAP2A or HIF‐1α/ΗΙF‐2α were silenced (by using specific siRNAs) in HeLa cells, following incubation under normoxia or hypoxia and mRNA levels were quantified. Verification of TFAP2A and HIF‐1α/HIF‐2α silencing is shown in Fig. 3A. In control cells (treated with control siRNAs‐si nt) grown under hypoxia, mRNA expression of GAPDH, LDHA, ENO1, TGFBI, SERPINE1, and ALDOC was significantly increased compared to normoxia (Fig. 3B), but was strongly impaired by HIF‐1α/ΗΙF‐2α silencing as anticipated by their previously established status of HIF‐target genes [11, 12, 22, 23, 24]. Interestingly, silencing of TFAP2A under normoxic conditions did not detectably affect the basal mRNA expression of the six tested genes, but inhibited significantly their expression under hypoxia at levels comparable with HIF‐1/2α silencing. However, silencing of TFAP2A under hypoxia did not affect mRNA expression of two other hypoxia‐dependent genes tested, like LOX‐1 and ITGA5 [25, 26] (Fig. 3B). Notably, silencing of TFAP2A did not influence transcriptional activation of genes (like RANTES, [27]) by TNFα. These results demonstrate the positive involvement of TFAP2A in the transcriptional activation of a subset of HIF‐dependent genes and not a generic impact on transcription.

### TFAP2A is necessary for HIF‐1 binding and chromatin accessibility in promoters of hypoxic genes

**Fig. 4:**
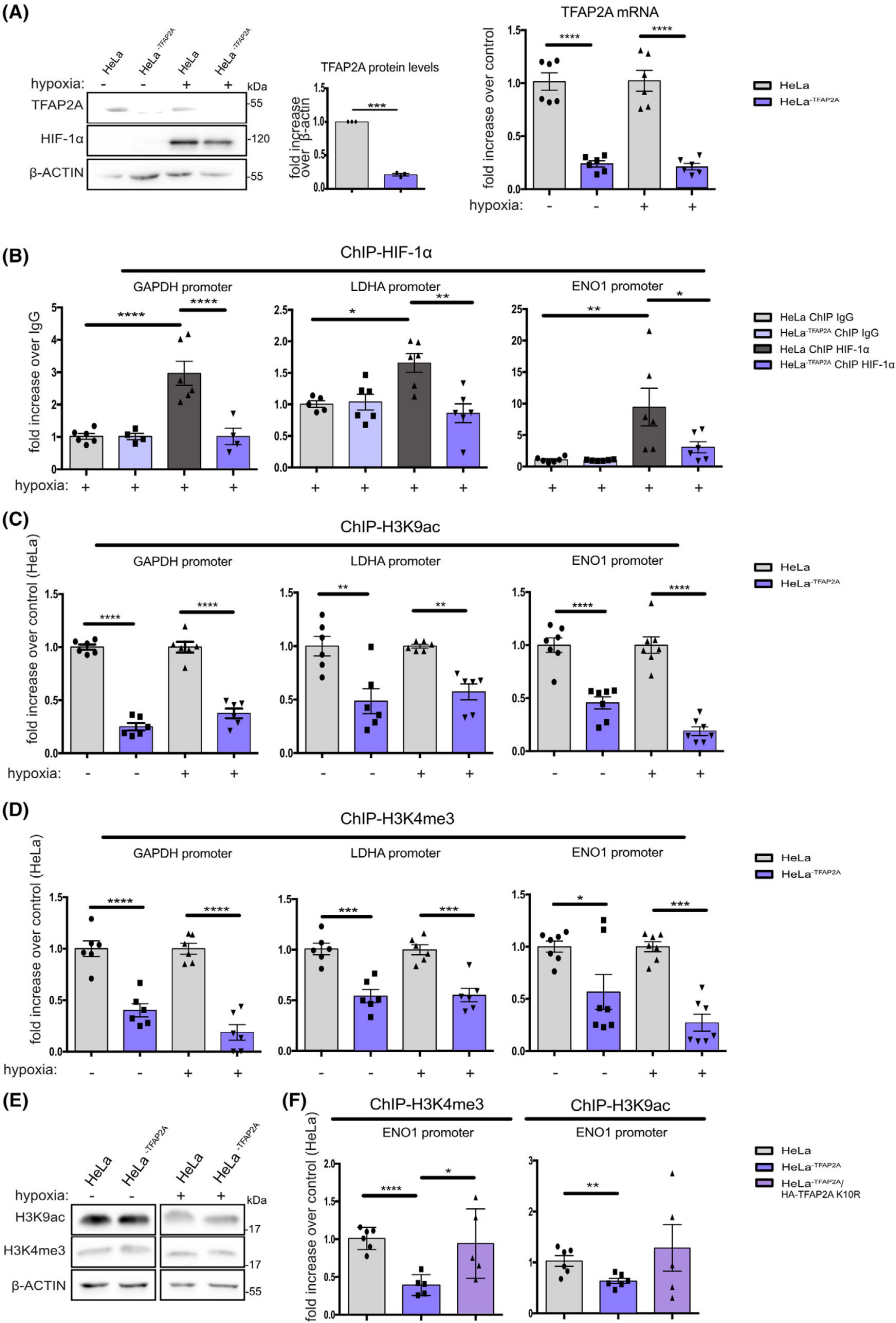
TFAP2A is necessary for HIF‐1 binding and chromatin accessibility in promoters of hypoxic genes. (A) HeLa and HeLa^−TFAP2A^ cells (CRISPR‐mediated knockdown for TFAP2A) were incubated in normoxia (−, 21% O_2_) or hypoxia (+, 1% O_2_) for 24 h. Analysis of TFAP2A and HIF‐1α protein levels by immunoblotting was performed using the indicated antibodies (left upper panel). Quantification of TFAP2A normoxic protein levels as mean from three independent experiments is shown. Determination of TFAP2A mRNA levels from the two cell lines used is shown at the right. Values are the mean of three independent experiments performed in duplicates 18S rRNA and RPLP1 were used for normalization. (B–D) HeLa wt and HeLa^−TFAP2A^ cells were incubated as in (A) and ChIP against HIF‐1α (B) or H3K9Ac (C) and H3K4me3 (D) was performed followed by qRT‐PCR analysis, using primers for the indicated promoters. ChIP data (B–C–D) are the mean of three independent experiments performed in duplicates −/+ SE. (E) Immunoblotting analysis of total HeK9ac and H3K4me3 levels in normoxia and hypoxia in Hela and Hela^−TFAP2A^ cell lines using the indicated antibodies. For the western blots, β‐actin was used as loading control. Position of MW is shown on the right. (F) HeLa wt, HeLa^−TFAP2A^, and HeLa^−TFAP2A^/HA‐TFAP2A K10R cells were incubated as in (A) and ChIP against H3K4me3 and H3K9Ac was performed followed by qRT‐PCR analysis, using primers for the ENO1 promoter. The Ct (threshold cycle) of immunoprecipitated samples (with specific antibodies or IgG) were normalized to the Ct of total input DNA (DCt). Results obtained with specific antibodies were then normalized to the ones obtained with IgGs (DDCt). Enrichment in (B) is expressed as fold increase compared to each IgG. Enrichment in (C), (D), and (F) is expressed as fold increase compared to HeLa wt condition in normoxia or hypoxia, respectively (*P < 0.05; **P < 0.01; ***P < 0.001, ****P < 0.0001, comparisons were made by one‐way ANOVA Tukey's multiple comparisons or *t*‐test (F)).

Having shown that TFAP2A can bind to a subset of hypoxia‐induced genes under both normoxia and hypoxia and can positively affect their hypoxia‐inducible transcription, we next addressed the question whether TFAP2A can facilitate binding of HIFs to chromatin. For this reason, we used CRISPR technology to create HeLa cell lines in which TFAP2A expression was drastically reduced (TFAP2A knock‐down; KD) as verified by both qPCR and western blotting (Fig. 4A). We were unable to create a complete knockout of TFAP2A as even its limited expression appeared to be essential for cell viability. We further verified that HIF‐1α levels remained the same in both cell lines tested under hypoxia (Fig. 4A). Using ChIP experiments, we could show that the enrichment of HIF‐1α in the promoters of GAPDH, LDHA, and ENO1, three genes with the strongest enrichment for TFAP2A together with HIF‐1α (Fig. 2A,C), was significantly decreased in HeLa^−TFAP2A^ cells expressing only residual levels of TFAP2A (Fig. 4B).

The diminished chromatin binding of HIF‐1α observed under TFAP2A knockdown could be attributed to changes in TFAP2A‐dependent chromatin modification and/or accessibility. We, therefore, performed ChIP analysis using antibodies against H3K4Me3 or H3K9Ac that are marks of active/open chromatin sites. Interestingly, knockdown of TFAP2A in HeLa^−TFAP2A^ cells significantly reduced both H3K4Me3 and H3K9Ac marks in all three of the promoters containing HIF‐1 and TFAP2A binding sites (Fig. 4C,D) compared to wild‐type HeLa cells under both normoxia and hypoxia. Interestingly, total nuclear levels for both H3K4me3 and H3K9ac marks were not influenced by knockdown of TFAP2A both under normoxia and hypoxia (Fig. 4E). In order to confirm if this reduction in histone marks is due to the knockdown of TFAP2A and not due to a nonspecific effect, we tested a Hela^−TFAP2A^ strain that stably expresses a TFAP2A form (HeLa‐TFAP2A K10R, see also Fig. 6) that is active in terms of inducing HIF activity [13]. As shown in Fig. 4F, histone mark levels in the ENO1 promoter, that were reduced in HeLa^−TFAP2A^ cells, were restored to normal levels in Hela^−TFAP2A^/HA‐TFAP2A K10R cell line, showing that this effect is specifically attributed to TFAP2A. Taken together, our data suggest that TFAP2A could act as a pioneer transcription factor that is prebound on chromatin of hypoxia‐inducible genes already under normoxia in order to facilitate its reorganization in an active state that can be readily accessible to HIFs when hypoxia triggers their stabilization and activation.

### TFAP2A interacts with the Ku70/80 complex for transcriptional activation in a SUMO‐dependent manner

**Fig. 5:**
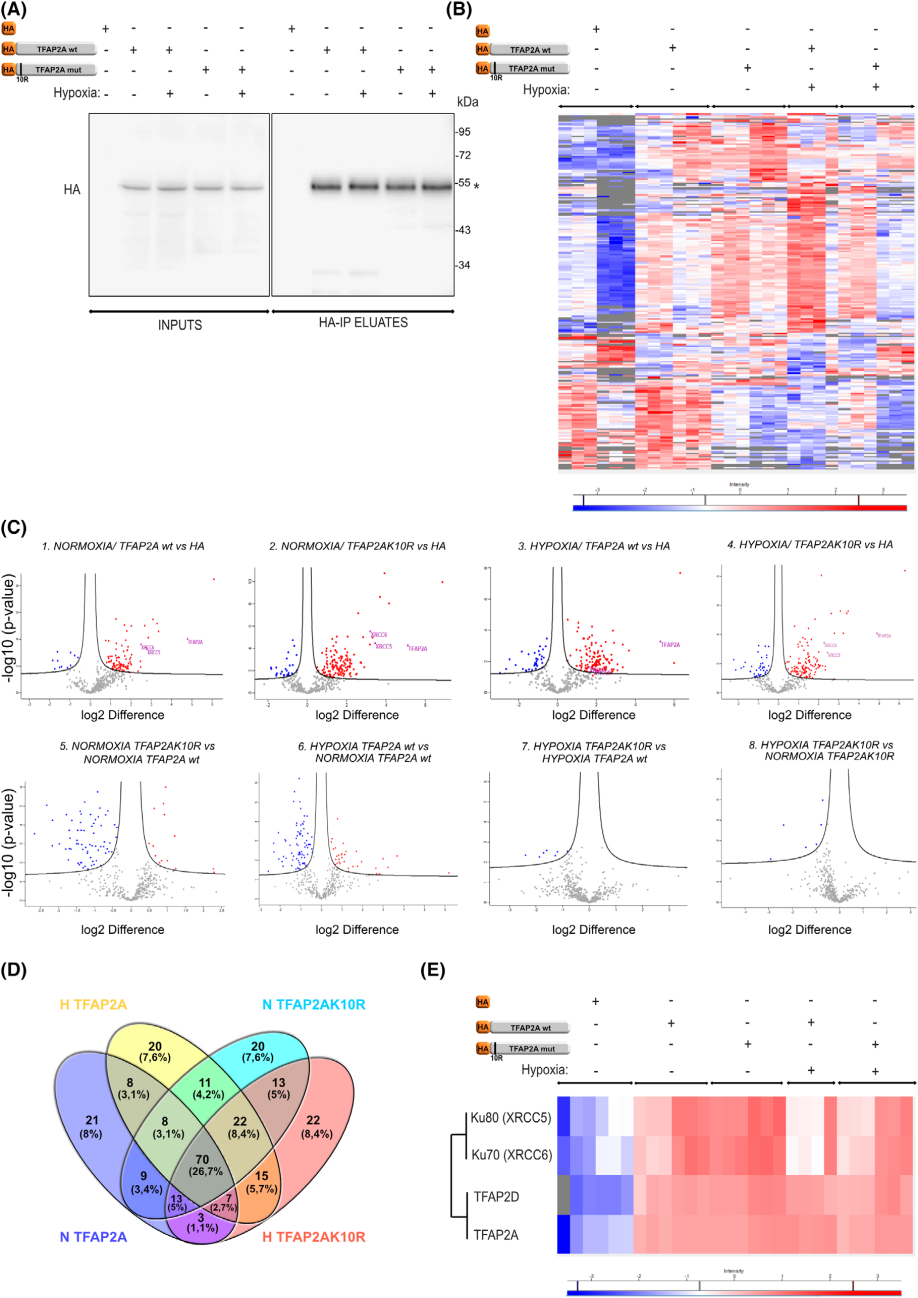
Interactome analysis between TFAP2A‐wt and the TFAP2AK10R mut in normoxia and hypoxia. HeLa cells overexpressing HA or HA‐TFAP2A wt and HA‐TFAP2AK10R mutant form were incubated in normoxia (−, 21% O_2_) or hypoxia (+, 1% O_2_) for 24 h and subjected to HA immunoprecipitation and proteomic analysis. (A) Immunoblotting analysis (representative of two biological replicates) of soluble extracts (INPUTS) or anti‐HA immunoprecipitates (HA‐IP ELUATES) using antibody against HA. Position of MW is shown on the right. (B) Heat map of z‐score intensities of all immunoprecipitated in the HA‐IP eluates from two biological repetitions analyzed in technical triplicates (except HA‐TFAP2A that a single analysis was performed for the second repetition, due to technical issues) in different conditions as described in (A). Color scale is shown below. (C) (Upper) Volcano plots of proteins exhibiting significantly differential binding to HA‐TFAP2A wt or HA‐TFAP2AK10R mutant forms compared to control (HA) under normoxia (N) or hypoxia (H) (#1‐#4). (Lower) Volcano plots of proteins exhibiting significantly differential binding to HA‐TFAP2AK10R compared to HA‐TFAP2A wt under normoxia (#5) or HA‐TFAP2A wt under hypoxia compared to HA‐TFAP2A wt in normoxia (#6) or HA‐TFAP2AK10R under hypoxia compared to HA‐TFAP2A wt under hypoxia (#7), or HA‐TFAP2AK10R under hypoxia compared to HA‐TFAP2AK10R under normoxia (#8). Red dots correspond to proteins with statistically increased enrichment; blue dots correspond to protein with statistically decreased enrichment; gray dots correspond to proteins with no statistical enrichment in the IP eluates. (D) Venn diagram representing the number of proteins preferentially bound to HA‐TFAP2A wt under normoxia (magenta), HA‐TFAP2A wt under hypoxia (yellow), HA‐TFAP2AK10R mut under normoxia (green), and HA‐TFAP2AK10R mut under hypoxia (pink). (E) Heat map of z‐score intensities of proteins Ku80 (XRCC5), Ku70 (XRCC6), TFAP2D, and TFAP2A (bait), immunoprecipitated in the HA‐IP eluates from two independent experiments in different conditions. Color scale is shown below.

To investigate further the involvement of TFAP2A in HIF‐dependent transcription, different HA‐tagged forms of TFAP2A were overexpressed in HeLa cells grown under normoxia or hypoxia and were immunoprecipitated with anti‐HA antibodies, in order to identify interacting proteins that may contribute to the role of TFAP2A. To examine the effect of TFAP2A SUMOylation on these interactions, in addition to the wild‐type form of HA‐TFAP2A (wt), we also used the TFAP2A K10R mutant form that cannot be SUMOylated and was shown previously to be more active than the wt form in stimulating the transcriptional activity of HIF‐1 [14]. Overexpression of HA epitope alone was used as specificity control. Similar expression and recovery between HA‐TFAP2A wt and HA‐TFAP2AK10R under both normoxia or hypoxia could be demonstrated by immunoblotting analysis of the cell lysates (INPUTS) and the anti‐HA immunoprecipitates (HA‐IP ELUATES) (Fig. 5A, HA‐TFAP2A wt and the K10R mut are marked with an asterisk). Subsequently, the ΗΑ‐IP eluates were processed by trypsin digestion and analyzed through liquid chromatography–tandem mass spectrometry (LC–MS/MS), which produced quantitative data for several hundred proteins in each IP eluate (Fig. 5B and File S4).

Out of the proteins that coprecipitated under normoxia, 139 exhibited specific and statistically significant enrichment in the HA‐TFAP2A wt IPs while 166 proteins were specifically recovered with the HA‐TFAP2A K10R mut IPs (Fig. 5C, volcano plot #1 and #2 and File S4), compared to control (HA). Accordingly, under hypoxia 161 proteins exhibited specific association with HA‐TFAP2A wt and 165 proteins with HA‐TFAP2A K10R mut (Fig. 5C, volcano plot #3 and #4 and File S4), compared to control (HA). From the identified proteins, a 26.7% is bound to all TFAP2A forms tested (Fig. 5D and File S4). Further comparison showed that there is a different pool of proteins that preferentially bind either to HA‐TFAP2AK10R (non‐sumoylated form) or HA‐TFAP2A wt under normoxia (a condition under which HA‐TFAP2A wt is normally SUMOylated) (Fig. 5D,C, volcano plot #5, statistically significant proteins shown in red and blue). In line with this, there is a distinct set of proteins that preferentially bind either to the HA‐TFAP2A wt under hypoxia (a condition under which HA‐TFAP2A wt loses its SUMOylation) or HA‐TFAP2A wt under normoxia (Fig. 5D,C, volcano plot #6). However, no major differences could be detected when we compared the set of interacting proteins between TFAP2AK10R mut and HA‐TFAP2A wt under hypoxia (a condition under which both TFAP2A forms are non‐SUMOylated) or between TFAP2AK10R mut under normoxia and the same protein under hypoxia (Fig. 5C, volcano plots #7 and #8). These results strongly suggest that the SUMOylation status of TFAP2A, and not hypoxia *per se*, is largely responsible for the observed differences in the interactome of TFAP2A under the different conditions tested.

Interestingly, proteins of the DNA‐PK (PRKDC) and DNA double‐strand break end‐joining complex such as Ku70 (XRCC6), Ku80 (XRCC5 or Ku86), were shown to be highly enriched in the IPs under all conditions tested, (Fig. 5C marked in purple, Fig. 5E and File S4). Ku70/Ku80 are part of the same ATPase helicase complex and are considered key components of the c‐NHEJ pathway [28] and regulatory subunits of the DNA‐PK catalytic subunit (DNA‐PKcs) [29] playing important roles in transcription. Interestingly, more proteins of this complex were found in our MS analysis such as the ILF2 and ILF3 (File S4). Furthermore, comparison of the proteomic data (Fig. 5E) showed that even though our bait (HA‐TFAP2A) and a known interactor of TFAP2A for heterodimerization, namely TFAP2D [16, 30] were found to be equally enriched in all conditions tested, Ku70/Ku80 associated preferably with HA‐TFAP2AK10R, that is, the SUMO‐deficient form, both under normoxia and hypoxia.

**Fig. 6:**
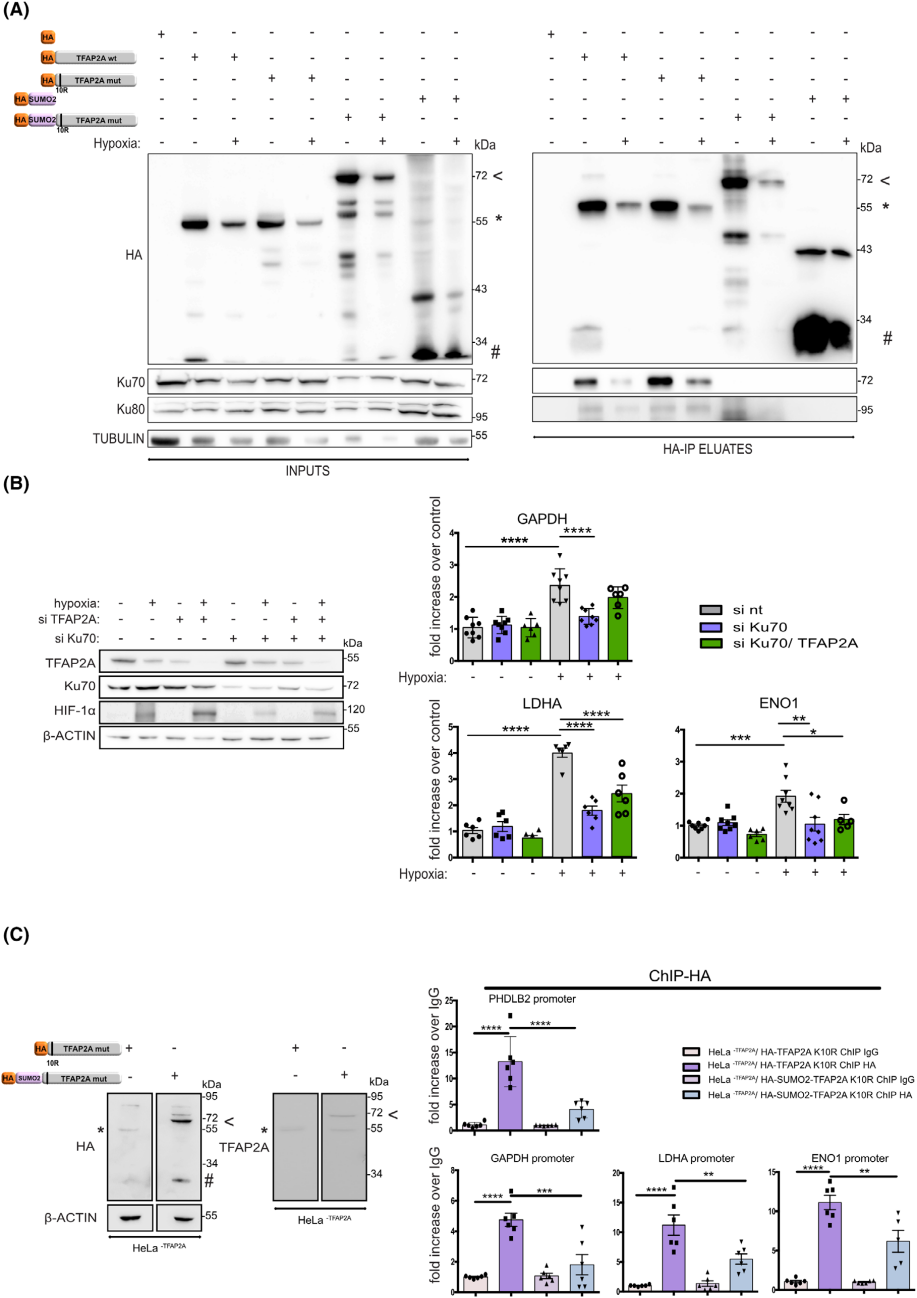
TFAP2A interacts with the Ku70/80 complex for transcriptional activation in a SUMO‐dependent manner. (A) HeLa cells overexpressing HA (neg. control), HA‐TFAP2A wt and K10R proteins or the HA‐SUMO2AA‐TFAP2AK10R form or HA‐SUMO2AA (neg. control), were incubated in normoxia (−, 21% O_2_) or hypoxia (+, 1% O_2_) for 24 and subjected to HA immunoprecipitation followed by immunoblotting analysis of soluble extracts (INPUTS) or anti‐HA immunoprecipitates (HA‐IP ELUATES), using the indicated antibodies. Position of MW is shown on the right. HA‐TFAP2A wt and the K10R mut are indicated with an asterisk (*), full‐length HA‐SUMO2AA‐TFAP2A K10R is indicated with an arrow (<) and free HA‐SUMO2AA is indicated with a hash (#). Tubulin was used as loading control and the western blot shown is representative of three independent experiments. (B) HeLa cells were transfected (48 h) with nt siRNA, or si against Ku70 and siRNA against both TFAP2A and Ku70 in normoxia and hypoxia (24 h). Immunoblotting analysis of the extracts (left) and determination of mRNA levels of the indicated genes, using qRT‐PCR was performed (right). PRLP1 rRNA was used for normalization. (C) HeLa^−TFAP2A^ cells stably expressing HA‐TFAP2AK10R or HA‐SUMO2AA‐TFAP2A K10R were incubated under normoxia for 24 h. Expression levels of the stably expressed proteins were observed by immunoblotting (left), using the indicated antibodies. ChIP against HA and qRT‐PCR analysis was also performed, using primers for the indicated promoters (right). Values in (B) and (C) are the mean of three independent experiments performed in duplicates and expressed as fold increase compared to each control conditions −/+ SE (si nt for (B), ChIP against each IgG for (C)) (*P < 0.05; **P < 0.01; ***P < 0.001, ****P < 0.0001, comparisons were made by one‐way ANOVA Tukey's multiple comparisons). For the immunoblot analysis, β‐actin was used as loading control and position of MW is shown on the right.

To verify the proteomic data and identify if the SUMO status of TFAP2A plays a role in its protein–protein interactions, the association of Ku70 with TFAP2A was analyzed by HA‐IP and western blotting both under normoxia and hypoxia (Fig. 6A and File S3 (Fig. S2A for repetition). In line with the MS analysis, we detected specific and almost equally strong association of Ku70 with both wild‐type and SUMO‐deficient forms of TFAP2A (Fig. 6A and File S3 (Fig S2B)   for quantification). As only a minor fraction of wild‐type TFAP2A is normally in the SUMOylated form [13], it is still possible that SUMOylated‐TFAP2A interacts less strongly with Ku70/Ku80 but this may not be easily detectable by comparing the wild‐type form to the SUMO‐deficient one. To better resolve this issue, we constructed and overexpressed HA‐SUMO2AA‐TFAP2A K10R, composed of a SUMO moiety fused directly to the N terminus of TFAP2A K10R, which imitates the fully SUMOylated form of TFAP2A. We could not detect Ku70 in the HA‐SUMO2AA‐TFAP2A K10R immunoprecipitates (Fig. 6A and File S3 (Fig S2)), suggesting that modification by SUMO weakens/abolishes the interaction between TFAP2A and Ku70. This also indicates that the physical association of TFAP2A with the Ku70/80 complex is stronger when the SUMO modification is removed under hypoxia and, therefore, may also implicate the Ku70/80 complex in the TFAP2A‐dependent transcriptional activation in response to hypoxia.

To test this hypothesis, we silenced Ku70 expression using specific siRNAs in HeLa cells under normoxia or hypoxia (Fig. 6B) and analyzed the mRNA expression levels of GAPDH, LDHA, and ENO1, three HIF and TFAP2A codependent genes also analyzed earlier (Figs 2, 3, 4). As shown in Fig. 6B, silencing of Ku70 expression decreased the mRNA expression levels of GAPDH, ENO1, and LDHA under hypoxia to a similar extent as observed in the case of TFAP2A silencing (Fig. 3B). Moreover, simultaneous silencing of both Ku70 and TFAP2A did not lead to a further reduction of mRNA levels of these genes (Fig. 6B), suggesting that Ku70 and TFAP2A collaborate as parts of the same complex/pathway in stimulating HIF‐dependent transcriptional upregulation.

To confirm the role of SUMO modification on TFAP2A function, we stably expressed the non‐SUMOylated (K10R mut) and the fully SUMOylated (SUMO2‐AA‐tagged‐TFAP2A K10R) form of TFAP2A in the Ηela^−TFAP2A^ CRISPR cell line (Fig. 6C) and performed ChIP against the HA epitope from cells grown under normoxia. Figure 6C shows that the TFAP2A K10R mut can efficiently bind to chromatin of a TFAP2A target gene, PHDLB2, as well as to HIF‐dependent genes such as GAPDH, ENO1, and LDHA, in a manner similar to endogenous wild‐type TFAP2A (shown in Fig. 2A). In contrast, the SUMO2‐AA‐tagged‐TFAP2A K10R exhibited a significantly lower ability to associate with the same promoters, suggesting that deSUMOylation of TFAP2A is not only necessary for efficient TFAP2A/Ku70 interaction but also for tight association of TFAP2A with the promoters of its target genes.

## Discussion

TFAP2A is considered as an important transcription factor for controlling both embryonic and oncogenic progression. Prompted by our previous findings that TFAP2A and its SUMOylation are involved in HIF‐dependent transcriptional activation [13], and in order to reveal the underlying mechanism, we employed genome wide profiling of TFAP2A binding to DNA under normoxic or hypoxic conditions. ChIP‐Seq and ChIP‐qPCR analysis allowed us to identify a subset of hypoxia‐regulated genes that are co‐occupied by both TFAP2A and HIF‐1α, both in HeLa and MCF7 cancer cell lines, providing evidence that binding of TFAP2A to HIF‐inducible promoters is not a cell type‐specific event. Interestingly, most of the genes identified as common TFAP2A and HIF targets are involved in glycolysis. That was a surprise as TFAP2A has never previously been associated with induction of glycolytic genes, despite the fact that it was associated with VEGF and angiogenesis [31].

Notably, TFAP2A exerted a positive influence on the expression of glycolytic enzymes such as GAPDH, LDHA, ENO1, and ALDOC, in cooperation with HIF‐1, since loss of TFAP2A repressed their hypoxia/HIF‐inducible mRNA expression. Moreover, TFAP2A could directly bind and stimulate promoters of genes involved in EMT and invasion such as TGFB1 and SERPINE1 that are also HIF‐related targets. It has been reported that TFAP2A can be involved in the EMT process in multiple ways, for example, by binding of TFAP2A to the TGFB1 promoter in HCCC cholangiocarcinoma cell lines [32, 33].

Our experiments also provide evidence that TFAP2A acts as a pioneer chromatin factor since it resides on the chromatin of hypoxic‐signature genes already under normoxia, where it may facilitate maintenance of a euchromatic active state. It has been long known that hypoxia‐inducible genes reside in open chromatin loci, preloaded with paused RNA pol II [5], so it is possible that TFAP2A is one of the factors required to make chromatin accessible to HIFs for at least a subset of their target genes. Consistent with this role, TFAP2A was shown to be associated with high levels of Histone H3 acetylation during development [34]. In the same vein, TFAP2A was also shown to counteract heterochromatin formation and enhance epigenetic activation of the E2F pathway promoters in melanoma cells [35]. Even though the mechanism behind this TFAP2A activity remains elusive, a recent study suggests that TFAP2A interacts with the nucleosome remodeling and deacetylase (NuRD) complex and prevents its deacetylation activity [35], which could explain its positive influence on euchromatin formation.

Our data now show that TFAP2A is not only needed for proper chromatin accessibility but also for orchestrating the assembly of hypoxia‐inducible transcriptional complexes. This is supported by our previous and present findings showing that TFAP2A physically interacts with HIF‐1 and HIF‐2 [13] as well as with Ku70/Ku80, regulatory subunits of DNA‐PK with multiple roles in transcription [36, 37]. Our study further provides evidence that TFAP2A is necessary for efficient interaction of HIF‐1 with its target genes, while Ku70 in complex with TFAP2A are also indispensable for optimum transcription of HIF‐1‐regulated genes. The Ku70/Ku80 complex was previously also shown to interact with TFAP2A in breast cancer cell lines [38], in which recruitment of Ku70 together with TFAP2A was needed for the transcriptional activation of the ERBB2 proximal promoter. More recently, HIF‐1α was shown to interact with TRIM28, DNA‐PKcs, Ku70, and Ku80 in MDA‐MB‐231 cells. Moreover, HIF‐1‐dependent recruitment of TRIM28 to HREs, along with DNA‐PK and Ku70 and Ku80, on a subset of hypoxia‐related genes was shown to be important for CDK9‐mediated activation of RNA pol II [39]. It is thus plausible that TFAP2A acts as a seed for assembly of a complex containing Ku70 and elements of the RNA polymerase II preinitiation complex that ensures basal level of expression of glycolytic and other hypoxia‐inducible genes already under normoxia, that is, in the absence of HIFs. Upon oxygen depletion and HIF activation, this complex could facilitate access and binding of HIFs to selected and functional HREs, thereby ensuring fast and maximal transcriptional activation in response to hypoxia.

**Fig. 7:**
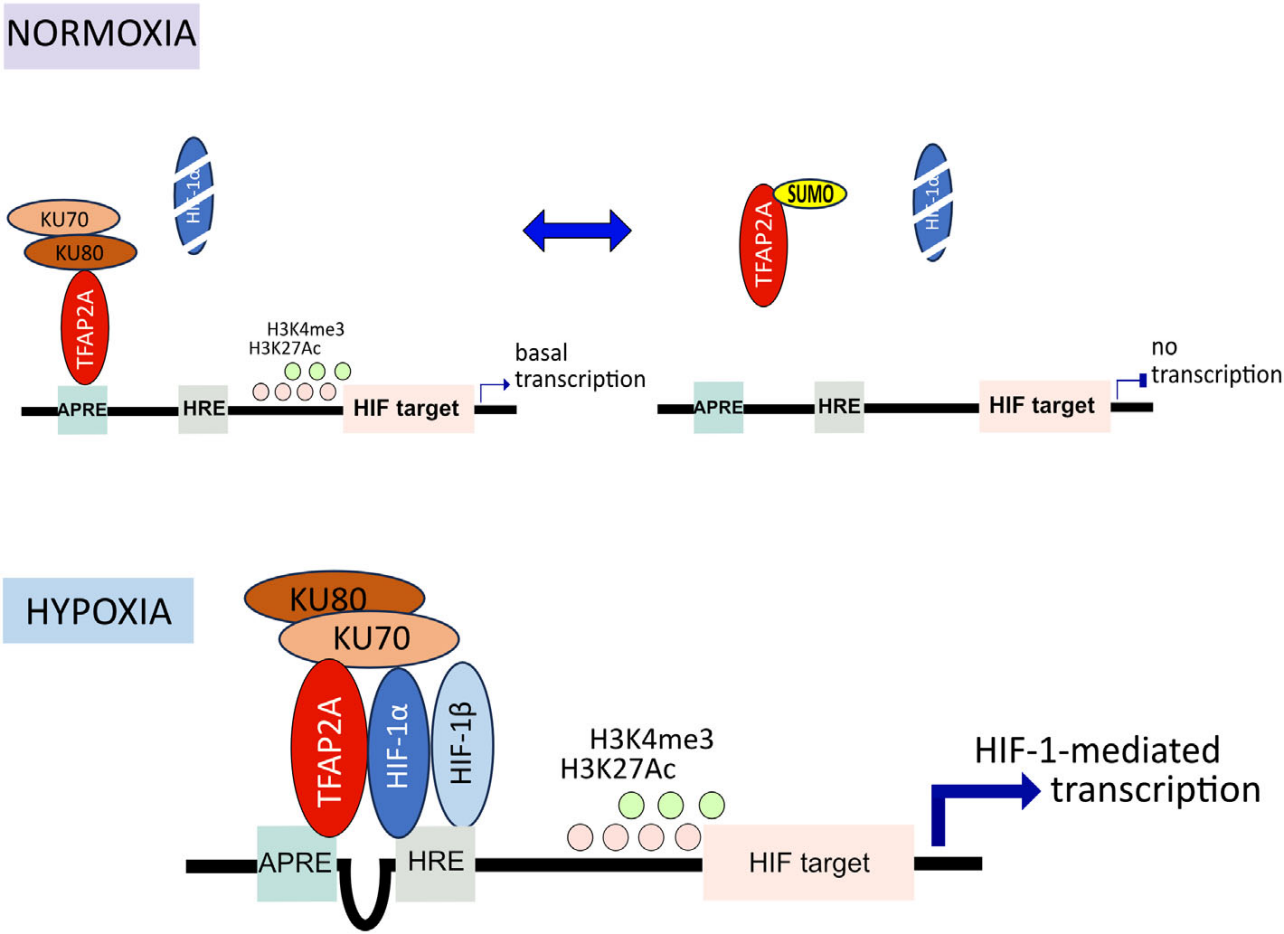
A proposed model on the role of the HIF‐1/TFAP2A complex during hypoxia. Under normoxia, TFAP2A is in an equilibrium of SUMOylated and unSUMOylated state. While the SUMOylated fraction of TFAP2A cannot interact with the Ku70/Ku80 complex nor it can efficiently bind to chromatin, unSUMOylated fraction of TFAP2A pre‐occupies chromatin of a subset of hypoxia‐induced genes together with Ku70/Ku80 complex and is necessary for basal deposition of H3K4me3 and H3K9Ac at promoter regions and euchromatin formation. Under hypoxia, the SUMOylation equilibrium shifts towards the unSUMOylated state of TFAP2A (hypoxia‐induced deSUMOylation of TFAP2A). This enhances its binding to DNA and its association with the Ku70/80 complex to ensure proper HIF binding, making chromatin more favorable for HIF‐1‐mediated transcription.

Dynamic SUMOylation provides another level of regulation and complexity in the HIF‐/TFAP2A‐axis. We and others have previously shown that TFAP2Α is SUMOylated by SUMO2/3 at position K10 [13, 40], which is close to the TFAP2Α transactivation domain (TAD). Although the initial trigger for hypoxia‐dependent deSUMOylation of TFAP2A is not yet known, we have provided evidence, using a non‐SUMOylatable and a fully SUMOylated form of TFAP2A, that its SUMO modification plays a dual role: It attenuates stable binding of TFAP2A to DNA and also inhibits its association with Ku70, thereby negatively affecting HIF‐dependent transcriptional activation (Fig. 7). SUMOylation can mitigate the activity of transcription factors in many ways [41, 42]. Therefore, extensive structural investigation of the TFAP2A‐Ku70 interaction is needed in order to understand the negative impact of SUMO. Since the SUMOylation site of TFAP2A resides at the N terminus close to its TAD, it is possible that addition of SUMO provides a steric hindrance that inhibits the TAD domain or other protein–protein interactions in the same area. Although SUMOylation occurs far from the DNA binding domain (DBD) and the helix‐span‐helix domain (HSH) (aa 203–420) of TFAP2A [16], it is also possible that bulk addition of SUMO2/3 or formation of SUMO2/3 chains could interrupt the binding of TFAP2A to the AP‐response element. (AP‐RE). We cannot also exclude that homodimerization of TFAP2A or heterodimerization with other members of the TFAP2 family could be negatively affected by SUMOylation.

In conclusion, our data demonstrate that TFAP2A is essential for HIF‐dependent activation of a subset of hypoxia‐inducible genes by mediating chromatin accessibility and recruitment of the Ku70/Ku80 complex at HRE and AP‐RE containing promoters. As TFAP2A loss leads to compromised hypoxic response with decreased expression of glycolytic and EMT‐related genes, targeting the TFAP2A/HIF interaction may offer a potent therapeutic approach for shutting down distinct routes of the HIF response pathway, which would in turn cause cancer cell death within hypoxic tumors.

## Materials and methods

### Plasmids

CMV‐AP2α wt containing the cDNA for the full‐length TFAP2A (isoform a) was kindly provided by Dr Bhattacharya (University of Oxford) [43]. pcDNA3.1‐HA‐TFAP2A wt and K10R mut were described before [13]. SUMO2 (GG) was cloned from pcDNA3‐cFOS‐SUMO2 (kindly provided by Guillaume Bossis) as BamHI‐EcoRI fragments to pcDNA3.1‐HA and pcDNA3.1‐HA‐TFAP2A K10R mut. PcDNA3.1‐HA‐SUMO2 (AA) and pcDNA3.1‐HA‐SUMO2 (AA)‐TFAP2A K10R mut was constructed with mutagenesis PCR using specific primers (File S3, Table S1) in order to change the last two amino acids of SUMO2 (GG to AA) so it will not be recognized and cleaved by endogenous SUMO‐specific proteases.

### Cell lines, transfection, and siRNA‐mediated depletion

HeLa cells (RRID:CVCL_0030, kindly provided by Dr F. Melchior, University of Heidelberg), and Human breast cancer MCF‐7 cells (RRID: CVCL_0031; kindly provided by Dr P. Moutsatsou, Medical School, Kapodistrian University of Athens, originally acquired from ATCC) were cultured in high glucose Dulbecco's modified Eagle's medium (Biosera, Nuaille, France) containing 10% heat‐inactivated (20 min, 56 °C) fetal bovine serum (FBS) (Gibco, Dublin, Ireland) and 100 U·mL^−1^ of penicillin–streptomycin antibiotic mix (P/S) (Biosera) in a CO_2_ humidified incubator (95% air and 5% CO_2_) at 37 °C. Cells were regularly tested for mycoplasma, and all experiments were performed with Mycoplasma‐free cells. For hypoxic experiments, cells were incubated in an INVIVO_2_ 200 hypoxia workstation (Baker Ruskinn, Sanford, ME, USA) at 1% O_2_, 94% N_2_, and 5% CO_2_. For TNFα experiments, cells were incubated with 10 μg·mL^−1^ TNFα for 4 h. Transient transfections with plasmid DNA were carried out in 10 cm, 6‐ or 12‐well plates by using a PEI (polyethylenimine) transfection reagent and were incubated for 24 h as previously described [13]. For siRNA‐mediated silencing, cells were transfected with 20 or 40 nm siRNAs using RNAiMax reagent (Invitrogen, Life Technologies, Carlsbad, CA, USA). Details on siRNAs are shown in File S3, Table S2.

### CRISPR–mediated gene knockdown

HeLa cells were transfected in a 60 mm culture dish with 1 μg of TFAP2A Double Nickase Plasmid (h) (Santa Cruz Biotechnology, Dallas, TX, USA) using jetPRIME® Polyplus reagent (Polyplus, Strasbourg, France). Cells were incubated in fresh DMEM with 10% FBS without P/S for 24 h. Transfected cells were detected using fluorescence microscopy and transferred in a 100 mm dish containing DMEM with 10% FBS with P/S. After 24 h, cells were incubated for 3 days in the presence of puromycin (Sigma‐Aldrich, St Louis, MO, USA) at a concentration of 2.5 μg·mL^−1^. Puromycin was removed, and transfected HeLa cells were grown for few days in order to form colonies of 50–100 cells each. A number of selected colonies were initially transferred in a 96‐well plate using cloning cylinders (Sigma‐Aldrich) and were further grown in order to be screened for mRNA and protein expression of TFAP2A.

### RNA isolation and quantitative real‐time PCR (qPCR)

RNA isolation and Quantitative Real‐time PCR were performed as previously described [44]. Specific primers (intron spanning) for the amplification of the target mRNAs were designed to generate amplicons of 70–100 bp with equal melting temperatures (See File S3, Table S3). Cycling conditions were 95 °C for 3 min, 40 cycles of 95 °C for 10 s, 60 °C for 20 s, and 72 °C for 1 s, followed by 95 °C for 5 s, 65 °C for 1 min, and 97 °C for 30 s. The primers used for the amplification of cDNAs for TFAP2A, LDHA, GAPDH, ENO1, ALDOC, TGFbI, SERPINE, LOX‐1, ITGA5, RANTES, RPLP1, and 18S are shown in File  S3 (Table S3). Each sample was assayed in duplicate for all targets and internal control. Relative quantitative gene expression was calculated using the ΔΔ*C*_T_ method, normalized to RPLP1 or 18S rRNA levels and is presented as a fold increase in relation to the appropriate control condition.

### Chromatin immunoprecipitation‐ and qPCR

HeLa and MCF7 cells after being incubated under normoxia or hypoxia for 24 h, were cross‐linked in 1% formaldehyde for 30 min, quenched with 0.125 m glycine for 5 min, and collected by centrifugation. The following steps were performed as previously described [45]. Antibodies for ChIP were used in a concentration of 2 μg per reaction (1.5–2 × 10^6^ cells) and are mentioned in File S3, Table S4. Amplification of regions containing TFAP2A binding sites relative to the promoter regions of PHLDB2, LDHA, GAPDH, ENO1, ALDOC, TGFBI, and SERPINE1 was performed in both immunoprecipitated and input samples using specifically designed primers listed in File S3, Table S3. The Ct (threshold cycle) of immunoprecipitated samples were normalized as previously described [25]. Results obtained with specific antibodies were then normalized to the ones obtained with IgGs (DDCt) and were expressed as fold increase over the IgG condition.

### Chromatin immunoprecipitation‐ and sequencing (ChIP‐seq), data analysis

HeLa cells were incubated under normoxia or hypoxia for 24 h, and TFAP2A with cross‐linked chromatin was immunoprecipitated as described above (Chromatin immunoprecipitation‐ and qPCR). Rabbit polyclonal anti‐TFAP2A (Thermo Fisher Scientific, PA5‐17359) was used in a concentration of 2 μg per reaction (1.5–2 × 10^6^ cells) (File S3, Table S4). Eluted chromatin from normoxic and hypoxic cells from two biological replicates were sequenced in an Ion Proton Sequencer.

The ChIP‐seq FASTQ files obtained after Ion Proton sequencing were first subjected to Quality Control using FASTQC (https://www.bioinformatics.babraham.ac.uk/projects/fastqc/) MultiQC (https://multiqc.info/). After QC, the FASTQ files were mapped against the hg19 assembly using bwa (http://bio‐bwa.sourceforge.net) with default settings. Peak calling was performed with macs [46].

For the analysis of the publicly available datasets, data from the ENCODE project were mined for TFAP2A ChIP‐seq (PRJNA63447). For the various HIF ChIP datasets, FASTQ files were downloaded from the PRJNA714954 dataset and were subsequently analyzed as described above. In all cases, the resulting BAM files were visualized in the UCSC Genome Browser using bedtools (https://github.com/arq5x/bedtools2) and tools provided by the UCSC Genome Browser toolkit. For ChIP‐seq peak annotation and Venn diagram plotting, the Bioconductor ChipSeeker (https://bioconductor.org/packages/release/bioc/manuals/ChIPseeker/man/ChIPseeker.pdf) [47] package was selected.

Bedtools intersect intervals were used for intersecting peak coordinates between replicates and factors. A first round of intersection was performed between the biological replicates of each factor, followed by a second round of intersection that included the consensus replicate peaks between different factor combinations. All intersections were performed with the following flags: ‐f 0.4, ‐F 0.4 ‐header. Intersected BED files were fed to ChIPSeeker for downstream peak annotation analysis and visualization. For the peak distance analysis, the ReldistBed tool from the bedtools suit [48] was used to calculate the relative peak distance between HIF1α and TFAP2A in normoxia or hypoxia separately for all target genes or only for the HIF1α targets based on the ChIPSeq peak annotation results. The peak distance was estimated on a scale from 0 to 0.5, where values close to zero indicate spatial correlation between two peak sets while values close to 0.5 indicate random peak association. Motif enrichment analysis was performed with Sea from the Meme suite [49] using the normoxia TFAP2 peaks as control against the Vertebrates motif database collection. Finally, all dot plots were performed with ggplot2 in r [50].

### Immunoblotting

Immunoblotting method was performed and analyzed as previously described [44] after analysis in 8–15% SDS/PAGE gels, used antibodies are presented in File S3, Table S4. Western blot images were taken by using a Uvitec Cambridge Chemiluminescence Imaging System supplied with alliance software (version 16.06) and quantified by uviband Software (version 15.03) supplied by the instrument manufacturer (Uvitec Cambridge, Cambridge, UK).

### Immunoprecipitation

For the anti‐HA nondenaturing immunoprecipitation HA‐TFAP2A, transfected cells were harvested with lysis buffer containing 150 mm NaCl, 50 mm Hepes‐NaOH pH 7,6, 1% Triton X‐100, 2 mm MgCl_2_, 5% glycerol, 1 mm AEBSF, and Protease Inhibitor Mix G (SERVA Electrophoresis GmbH, Heidelberg, Germany). Cell lysates were incubated with prewashed HA beads (10–15 μL bead volume per reaction) for 3 h at 4 °C under rotation. After thorough wash, bound proteins were eluted twice by incubation at 25 °C for 15 min with an excess of HA epitope‐spanning peptides (200 μg·mL^−1^ in lysis buffer from a 10 mg·mL^−1^ stock) or with SDS sample buffer (50 mm Tris pH 6.8, 2% SDS, 0.1% bromophenol blue, 10% glycerol). Immunoprecipitates were analyzed by mass spectrometry or SDS/PAGE followed by western blotting.

### Proteomic analysis

Proteomic analysis was performed according to previously published protocols [51, 52] and is analytically described below.

#### Protein digestion

The proteins eluted from the HA immunoprecipitation reactions were digested with trypsin using the Sp3 protocol [53]. Initially, the proteins were reduced and alkylated by DTT and Iodoacetamide. Then, magnetic bead mixture was added to the sample in 50% ethanol for the binding of the proteins onto the beads (mixture of hydrophilic and hydrophobic Sera‐Mag carboxylate‐modified beads, Cytiva, Marlborough, MA, USA). The samples were further cleaned‐up from impurities such as detergents and salt using a magnetic stand, through repeated washings with 80% ethanol and 100% acetonitrile. Finally, trypsin was added in order to digest the proteins overnight at 37 °C and with shaking at 1200 r.p.m. Next day, the peptide‐containing supernatants were aspirated and evaporated to dryness in a Speedvac.

#### LC–MS/MS

After solubilization, the separation of the peptides was carried out on a pepsep 25‐cm long nano column (1.9 μm 3 beads, 75 μm ID) for 1‐h long run. The mobile phases used were 0.1% (vol/vol) formic acid in water and 0.1% (vol/vol) formic acid in acetonitrile (ACN), starting with a gradient of 7% Buffer B to 35% Buffer B for 40 min and followed by an increase to 45% in 5 min and a second increase to 99% in 0.5 min for 5.5 min and then kept constant for equilibration at 7% Buffer B for 4.5 min.

The data acquisition was performed in positive mode using an Q Exactive HF‐X Orbitrap mass spectrometer (Thermo Fisher Scientific, Waltham, MA, USA)using a top12 DDA method. The resolving power was set to 12 K, and the maximum injection time was set to 100 ms. HCD MS/MS spectra were acquired with a target value of 1 × 10E5 and resolution of 15 K with a collision energy (NCE)of 28 and with maximum injection time for MS/MS set to 22 ms. Dynamic exclusion was enabled for 30 s. Peptide match was set as preferred. Three technical replicas were acquired per biological replicate.

#### Data analysis

The generated raw files were searched using the maxquant Software (2.0.2.0) [54] using Andromeda, against the *Homo sapiens* UniProt reference database downloaded (16/4/2021) and supplemented with a common contaminant database. The acceptable error was less than 20 ppm (first recalibration search). Up to 2 missed cleavages were allowed and the modifications considered were as follows: Oxidation (M); Acetyl (Protein N‐term); Deamidation (NQ) as variable modifications and Carbamidomethylation (Cys) as fixed modifications. Matching between runs and second peptide options were activated. The False discovery rate was set to 1% for protein, peptide. The label minimum ratio count was set to 2.

#### Data visualization

The LFQ maxquant search engine results were analyzed using perseus computational framework (version 1.6.15.0) [55]. The maxquant protein group search engine results were log2 transformed, filtered for potential contaminant, reversed hits and those only identified by site. The biological and technical replicates were grouped into two groups depending on if the IP was specific for the bait or for the control. The two groups were filtered fora minimum of 70% valid values in at least in one of the two groups. Remaining empty values were imputed based on normal distribution using default parameters in perseus. The groups were compared using a Student *t*‐test using permutation‐based FDR calculation (s0: 0.1, FDR: 0.05). The results were visualized in the form of volcano plots and heat maps.

Deposition: The mass spectrometry proteomics data have been deposited to the ProteomeXchange Consortium via the PRIDE [56] partner repository with the dataset identifier PXD053747.

### Statistical analysis

Statistical differences were assessed using the graphpad prisma version 6 software (GraphPad Software, San Diego, CA, USA). Data are expressed as mean ± SEM. Differences were examined by applying a two‐tailed Student's *t*‐test (between two groups) or by one‐way analysis of variance—ANOVA (Tukey's comparison within multiple groups). *P* < 0.05 was considered statistically significant (*P < 0.05; **P < 0.01; ***P < 0.001; ****P < 0.0001; n.s.: not significant).

### Nomenclature

SUMO‐1 (Smt3C; P63165), SUMO2 (Smt3A; also known as SUMO3, P55854), and SUMO3 (Smt3B; also known as SUMO2, P61956).

## Conflict of interest

The authors declare no conflict of interest.

## Author contributions

AK, CF, AnK, and GC performed experimental research, GC designed research and supervised experiments, GC, GS, and MS, contributed reagents/analytic tools; GSt performed MS analysis, MS and GP supervised MS analysis AG performed bioinformatic analyses, GC wrote the paper; AG, MS, and GS helped writing the paper.

## Supporting information


**File S1.** Total peak annotation report.

**File S2.** Sea results.

**File S3.** Supporting information (supplementary figures and tables).
**Fig S1.** Comparative peak analysis and Peak relative distance analysis.
**Fig S2.** SUMOylation status of TFAP2A affects interaction with Ku70 and its binding to DNA
**Table S1.** List of the DNA primers used in this study.
**Table S2.** List of siRNAs used in this study
**Table S3.** List of primers for RT‐PCR and ChIP‐qPCR analysis used in this study
**Table S4.** List of the antibodies used in immunoblotting and ChIP experiments.

**File S4**. Proteomics results.

## Data Availability

The mass spectrometry proteomics data have been deposited to the ProteomeXchange Consortium (http://proteomecentral.proteomexchange.org) via the PRIDE partner repository [56] with the dataset identifier PXD053747. The ChIP‐seq datasets are deposited in Gene Expression Omnibus (GEO) database [http://www.ncbi.nlm.nih.gov/geo/] under the accession code GSE271695.
