# Supplementary material for: Interaction of TFAP2A with the Ku70/80 complex is crucial for HIF‐dependent activation of hypoxia‐inducible genes
Source: FEBS J. 2025 Feb 24;292(16):4333–52. doi: 10.1111/febs.70025 (PMC12366265; doi:10.1111/febs.70025)
Supplement: Supplementary file 3 — File S3. Supporting information (supplementary figures and tables). Fig S1. Comparative peak analysis and Peak relative distance analysis. Fig S2. SUMOylation status of TFAP2A affects interaction with Ku70 and its binding to DNA Table S1. List of the DNA primers used in this study. Table S2. List of siRNAs used in this study Table S3. List of primers for RT‐PCR and ChIP‐qPCR analysis used in this study Table S4. List of the antibodies used in immunoblotting and ChIP experiments. [file FEBS-292-4333-s003.pdf]

Supporting information:

Supplementary Figures

Fig. S1: Comparative peak analysis and Peak relative distance analysis.

Fig. S2: SUMOylation status of TFAP2A affects interaction with Ku70 and its binding to DNA.

Supplementary Tables

Table S1: List of the DNA primers used in this study.

Table S2: List of siRNAs used in this study.

Table S3: List of primers for RT-PCR and ChIP-qPCR analysis used in this study.

Table S4: List of the antibodies used in immunoblotting and ChIP experiments.

Supplementary Figure S1

A

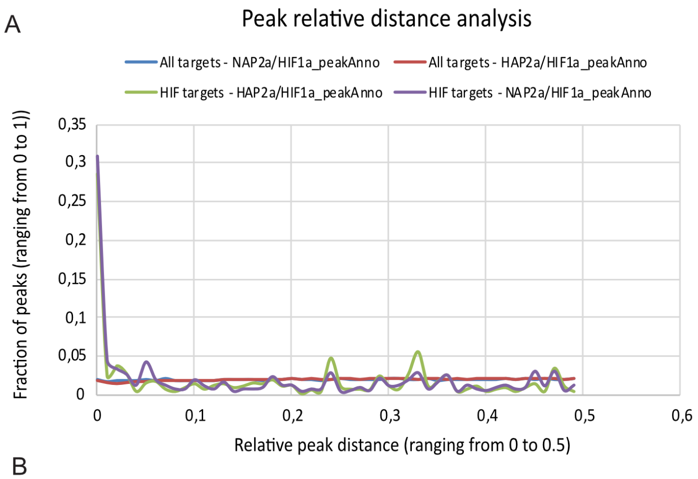

B

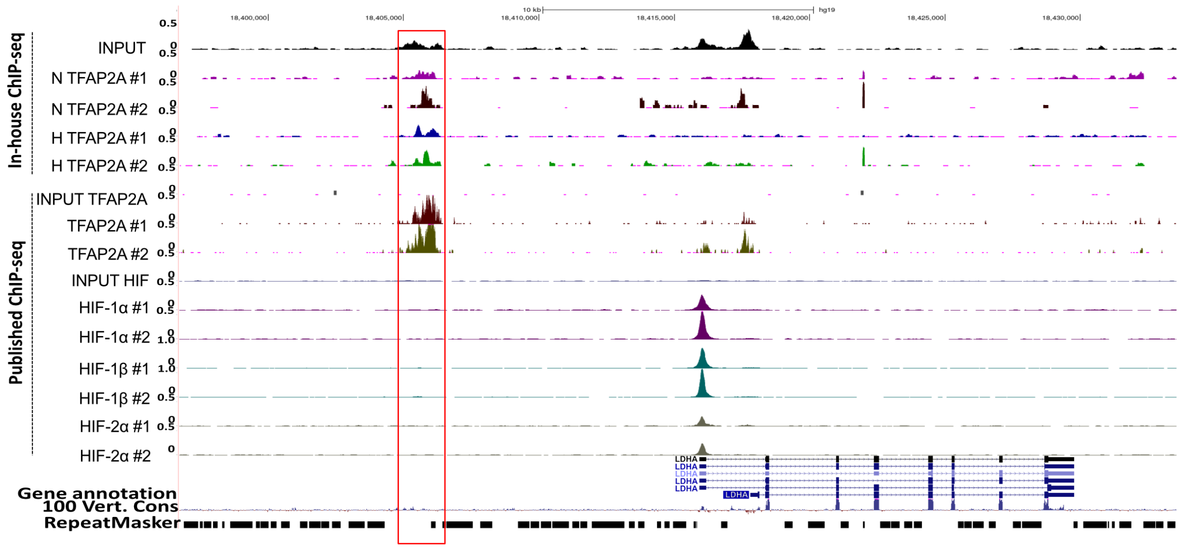

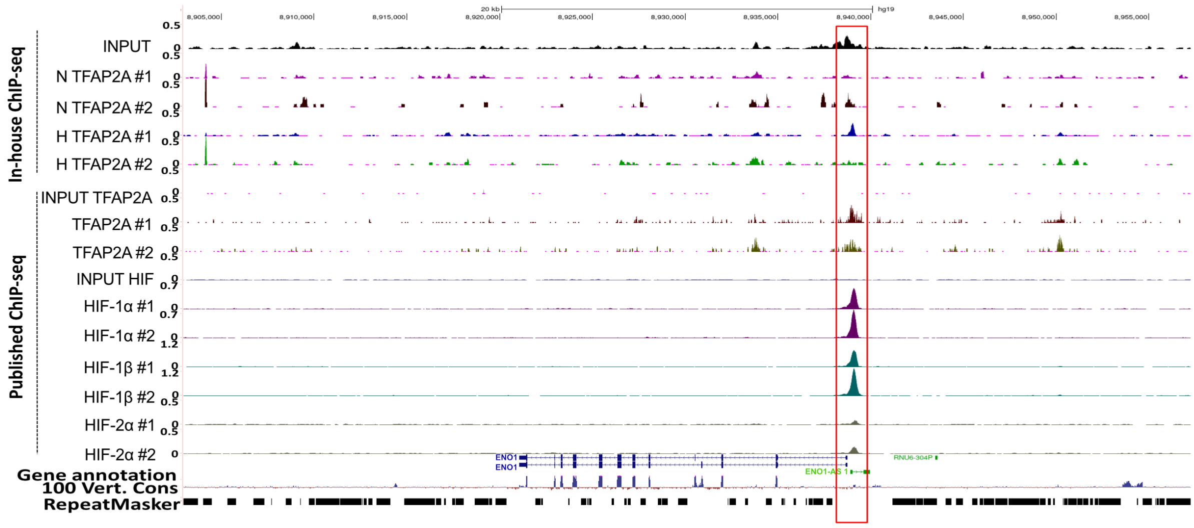

Supplementary Fig. S1: Peak relative distance analysis and comparative peak analysis. A. Peak relative distance analysis (presented on a scale between 0-0.5 with values close to 0 indicating spatial correlation of peaks) between the TFAP2A (from our own ChIP-Seq analysis) and the HIF peaks (from publicly available data [1] (PRJNA714954)) in the HIF-1 signaling pathway genes identified by our analysis (Fig 1E) to the pan-genomic relative distances.Y-axis indicates the fraction peaks (ranging from 0 to 1). B. Genome browser (https://genome.ucsc.edu) peak analysis on the promoters of ENO1 and LDHA genes and comparison of our in-house ChIP-seq data against TFAP2A, with publicly available ChIP-Seq against TFAP2A (ENCODE-PRJNA63447) and HIF-1α, HIF-2α, HIF-β in HeLa cells [1]. Peak of interest in each case is shown in a red frame.

References

1. Ortmann, B. M., Burrows, N., Lobb, I. T., Arnaiz, E., Wit, N., Bailey, P. S. J., Jordon, L. H., Lombardi, O., Peñalver, A., McCaffrey, J., Seear, R., Mole, D. R., Ratcliffe, P. J., Maxwell, P. H. & Nathan, J. A. (2021) The HIF complex recruits the histone methyltransferase SET1B to activate specific hypoxia-inducible genes, *Nat Genet.* **53**, 1022-1035.

Supplementary Figure S2

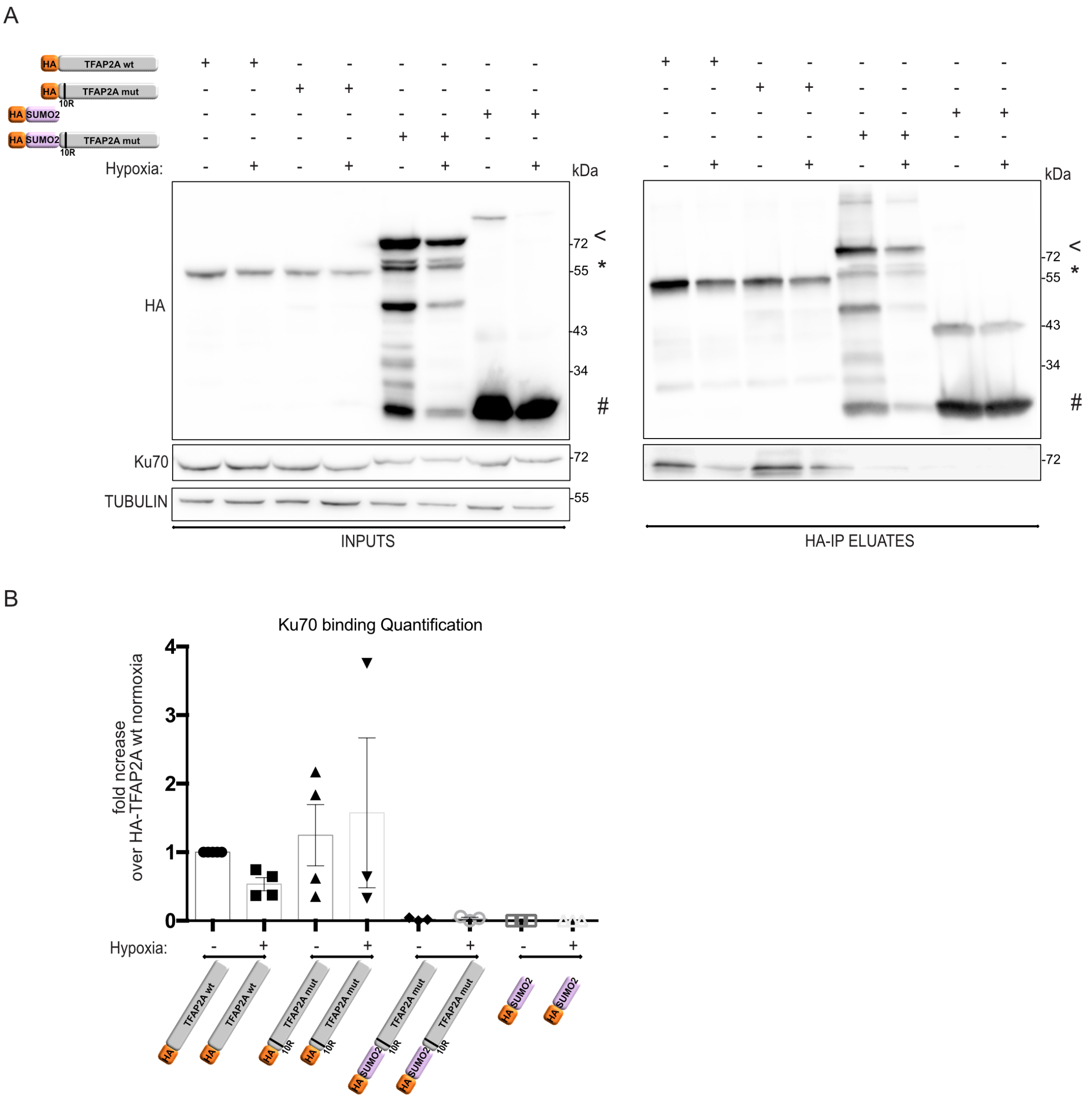

Supplementary Fig. S2: SUMOylation status of TFAP2A affects interaction with Ku70 and its binding to DNA. A. Immunoblotting analysis of soluble extracts (INPUTS) or anti-HA immunoprecipitates (HA-IP ELUATES) from HeLa cells overexpressing HA (neg. control), HA-TFAP2A wt and K10R proteins or the HA-SUMO2AA-TFAP2A K10R form and HA-SUMO2AA (neg. control), incubated in normoxia (-, 21% O_2) or hypoxia (+, 1% O_2) for 24 h, using the indicated antibodies. Position of MW is shown on the right. HA-TFAP2A wt and the K10R mut are indicated with an asterisk (*), full length HA-SUMO2AA-TFAP2A

K10R is indicated with an arrow (<) and free HA-SUMO2AA is indicated with a hash (#). Tubulin was used as loading control and the western blot shown is representative of three independent experiments. B. Ku70 and HA signals in the eluted samples, from at least 3 independent HA-IP experiments, were quantified and the ratio Ku70 /HA signal was calculated and expressed as fold increase compared to HA-TFAP2A wt in normoxia.

Supplementary Table S1.: List of the DNA primers used in this study

Oligonucleotide Name	*Sequence (5' – 3')*	*Application*	*Source*
SUMO2-AA-AP2A-Forward	GCAGCAGACGGCAGCTGAATTCATGCTTTGG	mutagenesis PCR SUMO2(GG) to SUMO2(AA)	Eurofins, LUX
SUMO2-AA-AP2A-Reverse	CCAAAGCATGAATTCAGCTGCCGTCTGCTGC	mutagenesis PCR SUMO2(GG) to SUMO2(AA)	Eurofins, LUX

Supplementary Table S2.: List of siRNAs used in this study

Oligonucleotide Name	*Sequence (5' – 3')*	*Source*
AllStars Negative Control siRNA	Proprietary	Qiagen,Venlo,Netherlands
TFAP2A siRNA	CAGCTCCACCTCGAAGTACAA	Qiagen, Venlo, Netherlands
HIF-1α siRNA	AGGAAGAACTATGAACATAAA	Qiagen, Venlo, Netherlands
HIF-2α siRNA	CCCGGATAGACTTATTGCCAA	Qiagen, Venlo, Netherlands
Ku70 (XRCC6) siRNA	ACCGAGGGCGATGAAGAAGCA	Qiagen, Venlo, Netherlands

Supplementary Table S3.: List of primers for RT-PCR and ChIP-qPCR analysis used in this study

Oligonucleotide Name	*Sequence (5' – 3')*	*Application*	*Source*
TFAP2A For	GACCTCTCGATCCACTCCTTAC	DNA primer for mRNA-qPCR analysis	Eurofins, LUX
TFAP2A Rev	GAGACGGCATTGCTGTTGGACT	DNA primer for mRNA-qPCR analysis	Eurofins, LUX
RPLP1 For	AAGCAGCCGGTGTAAATGTTGAGC	DNA primer for mRNA-qPCR analysis	Eurofins, LUX

RPLP1 Rev	CATTGCAGATGAGGCTCCCAATGT	DNA primer for mRNA-qPCR analysis	Eurofins, LUX
LDHA For	CCAACATGGCAGCCTTTTCC	DNA primer for mRNA-qPCR analysis	Eurofins, LUX
LDHA Rev	ACCAGCTTGGAGTTTGCAGT	DNA primer for mRNA-qPCR analysis	Eurofins, LUX
GAPDH For	AGCCACATCGCTCAGACAC	DNA primer for mRNA-qPCR analysis	Eurofins, LUX
GAPDH Rev	GCCCAATACGACCAAATCC	DNA primer for mRNA-qPCR analysis	Eurofins, LUX
ENO1 For	GGGTTTGCTCCCAACATCCT	DNA primer for mRNA-qPCR analysis	Eurofins, LUX
ENO1 Rev	CAGTGTAGCCAGCTTTCCCA	DNA primer for mRNA-qPCR analysis	Eurofins, LUX
ALDOC For	CTGCCACTGAGGAGTTCATC	DNA primer for mRNA-qPCR analysis	Eurofins, LUX
ALDOC Rev	CTCCACCATCTTCTCCACTG	DNA primer for mRNA-qPCR analysis	Eurofins, LUX
TGFbI For	GTCCACAGCCATTGACCTTT	DNA primer for mRNA-qPCR analysis	Eurofins, LUX
TGFbI Rev	ACCGCTCACTTCCAGAGAGA	DNA primer for mRNA-qPCR analysis	Eurofins, LUX
SERPINE For	GAAAGGCAACATGACCAGGC	DNA primer for mRNA-qPCR analysis	Eurofins, LUX
SERPINE Rev	CATGCGGGCTGAGACTATGA	DNA primer for mRNA-qPCR analysis	Eurofins, LUX
18S For	CTCAACACGGGAAACCTCAC	DNA primer for mRNA-qPCR analysis	Eurofins, LUX
18S Rev	CGCTCCACCAACTAAGAACG	DNA primer for mRNA-qPCR analysis	Eurofins, LUX
LOX-1 For	CGA CGA CCC TTA CAA CCC CTA C	DNA primer for mRNA-qPCR analysis	Eurofins, LUX

LOX-1 Rev	AAA TCT GAG CAG CAC CCT GTG	DNA primer for mRNA-qPCR analysis	Eurofins, LUX
RANTES For	CCT CAT TGC TAC TGC CCT CT	DNA primer for mRNA-qPCR analysis	Eurofins, LUX
RANTES Rev	GGT GTG GTG TCC GAG GAA TA	DNA primer for mRNA-qPCR analysis	Eurofins, LUX
ITGA5 For	CTATGGCGTCCCACTGTGG	DNA primer for mRNA-qPCR analysis	Eurofins, LUX
ITGA5 Rev	GTGGCTGGAGGCTTGAGC	DNA primer for mRNA-qPCR analysis	Eurofins, LUX
ChIP-PHLDB2 For	GCTGTCTGGTGAGGAAATCAT	DNA primer for ChIP-qPCR promoter analysis	Eurofins, LUX
ChIP-PHLDB2 Rev	GCAACGCAGTAGGGACTTT	DNA primer for ChIP-qPCR promoter analysis	Eurofins, LUX
ChIP-LDHA For	GGAGGGAGCTAGGGAGTATTT	DNA primer for ChIP-qPCR promoter analysis	Eurofins, LUX
ChIP-LDHA Rev	TCACAGTCTGGGAGGATAGTAAG	DNA primer for ChIP-qPCR promoter analysis	Eurofins, LUX
ChIP-GAPDH For	CTCACCCTGCCCTCAATATC	DNA primer for ChIP-qPCR promoter analysis	Eurofins, LUX
ChIP-GAPDH Rev	CAGTGTGCCTTTCATTCCATC	DNA primer for ChIP-qPCR promoter analysis	Eurofins, LUX
ChIP-ENO1 For	CTCGACCTTGCTGACAACTT	DNA primer for ChIP-qPCR promoter analysis	Eurofins, LUX
ChIP-ENO1 Rev	AACGACTCGACGCCAAC	DNA primer for ChIP-qPCR promoter analysis	Eurofins, LUX
ChIP-ALDOC For	GTAAATGAGGCTGCGGATGT	DNA primer for ChIP-qPCR	Eurofins, LUX

		promoter analysis	
ChIP-ALDOC Rev	TCTGAGGGCGTGGTCTT	DNA primer for ChIP-qPCR promoter analysis	Eurofins, LUX
ChIP-TGFbI For	GGTTGTTGACTCACGAGATGA	DNA primer for ChIP-qPCR promoter analysis	Eurofins, LUX
ChIP-TGFbI Rev	GGAAGGAGAAAGTGGGTTCAA	DNA primer for ChIP-qPCR promoter analysis	Eurofins, LUX
ChIP-SERPINE For	GGCATGGCAGACAGTCAA	DNA primer for ChIP-qPCR promoter analysis	Eurofins, LUX
ChIP-SERPINE Rev	CAATAGCCTTGGCCTGAGAA	DNA primer for ChIP-qPCR promoter analysis	Eurofins, LUX

Supplementary Table S4.: List of the antibodies used in immunoblotting and ChIP experiments.

Antibody/Cat.number	*Dilution*	*Reference/Source*
Rabbit polyclonal anti-HA-tag / C29F4	WB: 1:1000 or 1:2500	Cell Signaling, Massachusetts, USA
Rabbit polyclonal anti-HIF1α	WB: 1:250-1:1000<br>ChIP: 2μg/reaction	[1]
Rabbit monoclonal anti-TFAP2A/ MA5-14856	WB: 1:1000<br>ChIP: 2μg/reaction	Invitrogen, Life Technologies, Carlsbad, CA, USA
Rabbit monoclonal anti-Ac-H3 (Lys9)/ C5B11,	WB: 1:1000<br>ChIP: 2μg/reaction	Cell Signaling, Danvers, MA, USA
Rabbit Tri-Methyl-Histone 3 (Lys 4) / C42D8	WB: 1:1000<br>ChIP: 2μg/reaction	Cell Signaling, Danvers, MA, USA
Mouse monoclonal antiHIF-1α/ 610959	WB: 1:500	BD Transduction Laboratories (New Jersey, USA)
Rabbit polyclonal anti-HIF-2α/ NB100- 122	WB: 1:2000	Novus Biologicals, Cambridge, UK

Mouse monoclonal anti-β-Actin/ 3700S	WB: 1:5000	Cell Signaling, Danvers, MA, USA
Mouse monoclonal anti-α-tubulin/ 3873S	WB: 1:10000	Cell Signaling, Danvers, MA, USA
Rabbit monoclonal anti-Ku70/ D10A7	WB: 1:1000	Cell Signaling, Massachusetts, USA
Rabbit monoclonal anti-Ku80/ C48E7	WB: 1:1000	Cell Signaling, Massachusetts, USA
Secondary Goat Anti-Rabbit HRP	WB: 1:5000	Cell Signaling, Massachusetts, USA
Secondary Horse Anti-Mouse HRP	WB: 1:5000	Cell Signaling, Massachusetts, USA

1. Lyberopoulou, A., Venieris, E., Mylonis, I., Chachami, G., Pappas, I., Simos, G., Bonanou, S. & Georgatsou, E. (2007) MgcRacGAP interacts with HIF-1alpha and regulates its transcriptional activity, *Cell Physiol Biochem.* **20**, 995-1006.
